# Characterization and properties of micro- and nanowires of controlled size, composition, and geometry fabricated by electrodeposition and ion-track technology

**DOI:** 10.3762/bjnano.3.97

**Published:** 2012-12-17

**Authors:** Maria Eugenia Toimil-Molares

**Affiliations:** 1 Materials Research Department, GSI Helmholtz Centre for Heavy Ion Research, Planckstr. 1, 64291 Darmstadt, Germany

**Keywords:** electrodeposition, etched ion-track membrane, finite-size effects, heavy ion irradiation, nanowire, radiation-induced nanostructures

## Abstract

The combination of electrodeposition and polymeric templates created by heavy-ion irradiation followed by chemical track etching provides a large variety of poly- and single-crystalline nanowires of controlled size, geometry, composition, and surface morphology. Recent results obtained by our group on the fabrication, characterization and size-dependent properties of nanowires synthesized by this technique are reviewed, including investigations on electrical resistivity, surface plasmon resonances, and thermal instability.

## Introduction

During the past decade, nanowires have attracted an enormous interest due to a large variety of promising applications in areas such as nanoelectronics, biotechnology, magnetism, thermoelectrics, solar cells, and water splitting, among others [1–4]. Their reduced size, elongated geometry, and high surface-to-volume ratio turn nanowires into ideal elements for electrical and electrochemical systems [5–6]. In addition, nanowires are considered excellent model objects to study how fundamental physical properties (such as mechanical, optical, electronic, thermoelectrical and magnetic) depend on dimension, composition, geometry and crystallinity of the nanostructures [7–9]. The investigation of size effects requires methods to synthesize nanowires under controlled conditions and with tailored characteristics. Moreover, to characterize physical and chemical properties at the single-nanowire level requires appropriate techniques. In the two areas of fabrication and characterization, great advances have been reported in recent years. Methods to fabricate nanowires include top-down approaches such as optical and electron-beam lithography, and focused ion beam. More commonly applied bottom-up approaches are, e.g., vapour–liquid–solid growth, sol–gel and other chemical methods [10–11]. This review focuses on the bottom-up template method, which provides nanowires of a great variety of materials, from metals to semiconductors, including polymers as well as inorganic and organic compounds [12]. The material of interest is synthesized in the channels or cavities of the given template. During growth, the nanostructures adopt the exact shape and size of the hosting channels [13]. The most commonly used templates are porous alumina [14], diblock-copolymers [15], and track-etched membranes. Electrochemical and electroless deposition, polymerisation reactions, sol–gel template synthesis, and high-pressure injection of a melted material are examples of available techniques suitable for filling the pores. The electrodeposition of 40 nm diameter metal nanowires (Sn, In, and Zn) in etched fission tracks in mica was reported by Possin et al. back in 1970 [16]. In 1984, Williams and Giordano employed the same method to synthesize nanowires with a diameter as small as 10 nm using mica templates [17]. Since then, a large variety of materials have been electrodeposited, mainly in polymeric etched ion-track membranes [18–24]. Advantages of the electrodeposition method include low fabrication cost, high deposition rates, and its suitability for filling low- and high-aspect-ratio pores and trenches [25]. The wires are grown from the bottom to the top, yielding homogeneous replication of channels with any given geometry [26]. All relevant parameters, such as wire diameter, wire density, geometry, material and crystallinity, can be adjusted, allowing systematic studies of finite- and quantum-size effects on wire properties relevant for various technological applications.

This paper reviews recent advances in the electrodeposition of metal, semimetal, and semiconductor nanowires in polymeric etched ion-track membranes. Particular focus is given to our current efforts to study the influence of size, morphology and crystallinity of nanowires on electrical, optical and thermal properties. In section 1, we discuss the processes involved in the fabrication of etched ion-track membranes and electrodeposition of nanowires. Section 2 includes results on the compositional and crystallographic characterization of nanowires of various materials including metals, semimetals and semiconductors. The different nanowire morphologies attained by deposition in etched ion-track membranes are summarized in section 3. Finally, in section 4, recent results obtained by our group on electrical, optical, and thermal size-effects of the electrodeposited nanowires are presented.

## Review

### 1 Nanowire fabrication

#### 1.1 Fabrication of etched ion-track membranes

In the past two decades, etched ion-track membranes have been widely used as templates for the creation of nanowires and nanotubes. Their fabrication involves two separate processing steps: (i) Irradiation of the template material with energetic heavy ions and creation of latent tracks; (ii) selective ion-track dissolution and formation of channels by chemical etching. Control over the irradiation and etching conditions enables the production of various membranes with channels of predefined geometries, sizes and aspect ratios.

**1.1.1 Swift heavy-ion irradiation:** Swift heavy-ion beams are provided at large accelerator facilities, such as the linear accelerator of GSI (Darmstadt, Germany), and the cyclotrons at GANIL (Caen, France), JINR (Dubna, Russia), and CICLONE (Louvain la Neuve, Belgium) and a few others outside Europe, for example in Lanzhou (China) and Brookhaven (USA). The UNILAC linear accelerator of GSI provides heavy ions (up to uranium) of specific energy up to 11.4 MeV per nucleon (MeV/u) corresponding to ≈15% of the velocity of light [27]. Ion beams of such high energy have a penetration range in polymers of about 120 µm. Given this large range, foil stacks (e.g., ten foils 12 µm thick, or four foils 30 µm thick) can be irradiated. Each ionic projectile induces electronic excitation and ionisation processes in a cylindrical zone along its trajectory. In polymers, chemical bonds are destroyed and small volatile fragments (e.g., H_2_, CO, CO_2_, hydrocarbons) easily outgas [28]. This damaged region is called the ion track and has a typical diameter of few nanometres.

**Figure 1:**
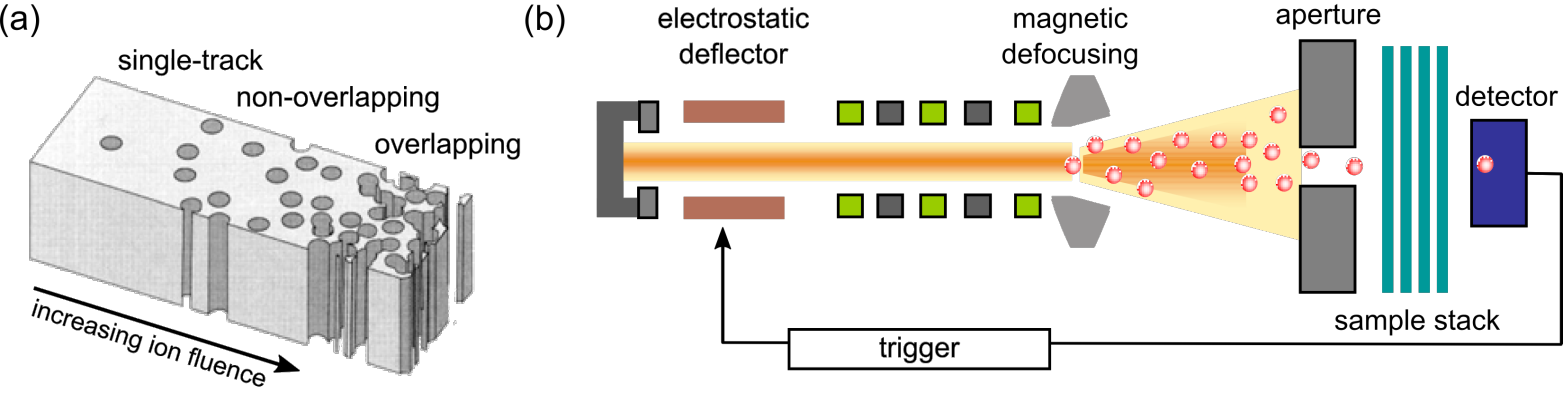
(a) Track-etched membrane illustrating the porosity regime available by means of ion-track technology: single channel, non-overlapping channels, and overlapping channels. (b) Schematic of single-ion irradiation setup.

By suitable adjustment of the ion beam and monitoring the flux (beam current), the applied ion fluence can be adjusted over a wide range, from exposure to a single ion (single track) up to more than 10^12^ ions/cm^2^ (overlapping tracks) (Figure 1a). At the UNILAC beamline of the GSI facilities, irradiation with a broad homogenous beam is obtained by magnetic defocusing. Samples of up to several square centimetres in size can be exposed. The resulting ion tracks are stochastically distributed and oriented in parallel across the sample. Irradiation with one single ion requires monitoring of individual ions hitting the sample [29]. To achieve this, the sample is irradiated through a small circular aperture (diameter ≈ 200 μm) placed in front of a stack of foils. The ion beam is strongly defocused and adjusted in such a way that single projectiles pass through the aperture with a frequency of about 1 Hz. The ions are detected by a solid-state particle detector placed behind the sample. As soon as the detector has registered a single ion impact, the entire ion beam is deflected by an electrostatic chopper system. A schematic representation of the single-ion irradiation system is presented in Figure 1b. This GSI single-ion irradiation facility is routinely used for the production of single-nanopore membranes [30–33] and the growth of single nanowires [34–36]. If required for specific applications, it is possible to create a preset regular ion-track arrangement by using a microprobe [37]. Materials commonly used as multi- and single-pore etched-ion-track membranes include polymers such as polyimide (PI), polyethylene terephthalate (PET) and polycarbonate (PC), and inorganic materials such as mica and glass.

**Figure 2:**
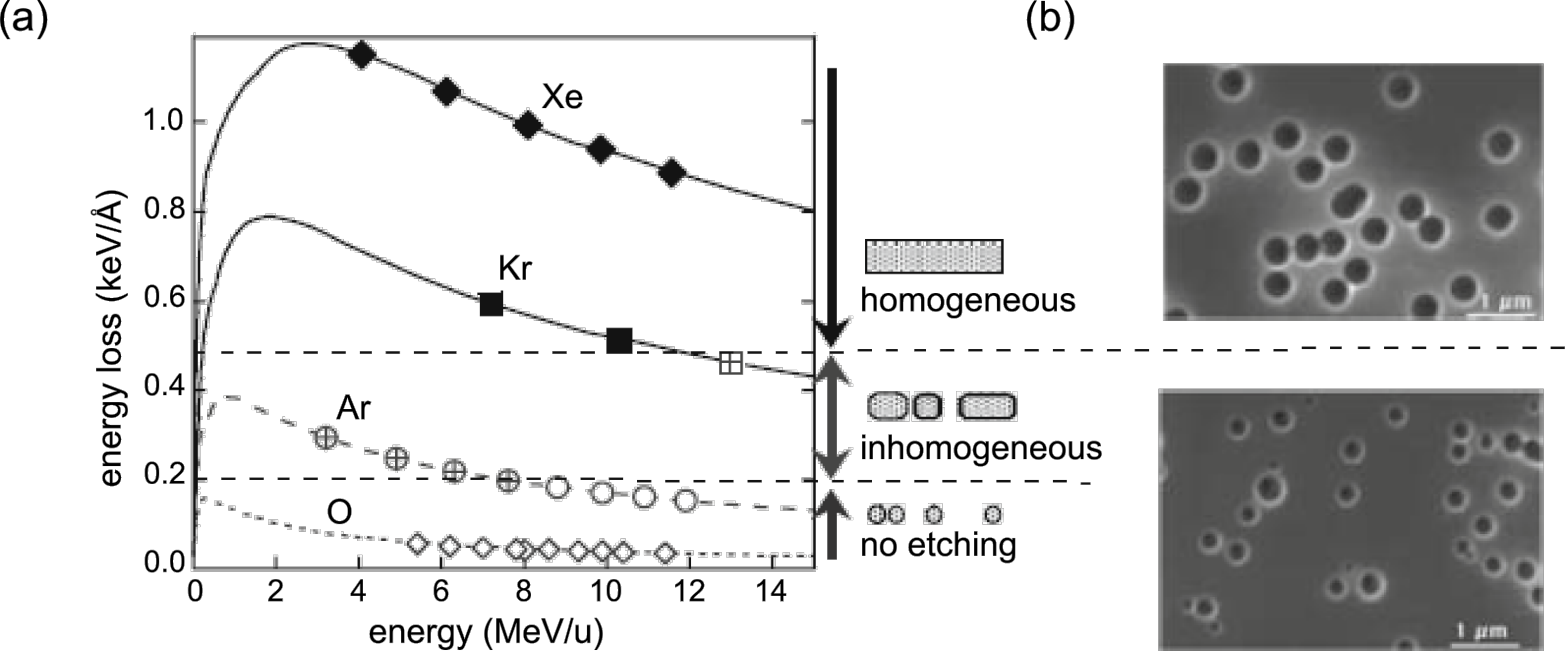
(a) Energy loss as a function of specific ion energy. The dashed lines separate the different regimes of track etching: homogenous (full symbols), inhomogeneous (crossed symbols), and absence of etching (open). (b) SEM images of uniform pores resulting from homogeneous tracks (top) and pores with broad size distribution due to inhomogeneous tracks (bottom). Adapted with permission from [38] – Copyright 1996 Elsevier.

The production of membranes with open channels requires selective dissolution of the latent tracks (cf. subsection 1.1.2). Selective track etching of channels with small size distributions requires continuous and homogeneous damage along the ion trajectory. Best results are achieved when the energy loss of the ions in the given material is above the so-called etching threshold [38]. Figure 2a presents the energy loss of light- and heavy-ion projectiles in polyimide. The different symbols denote cylindrical (full), discontinuous (crossed), and spherical (open) damage morphology with respective homogenous (full), inhomogeneous (crossed), and missing (open) selective track etching. Scanning electron microscopy (SEM) images in Figure 2b reveal how the etching of homogeneous tracks results in channels of uniform size (top) after etching tracks of homogeneous damage, whereas etching of inhomogeneous tracks leads to pores with a broad size distribution (bottom) [38]. Heavier projectiles (e.g., Au, Pb, Bi, U), produce tracks of more pronounced and continuous damage and are thus optimal for the production of porous membranes with small pore size distributions.

**Figure 3:**
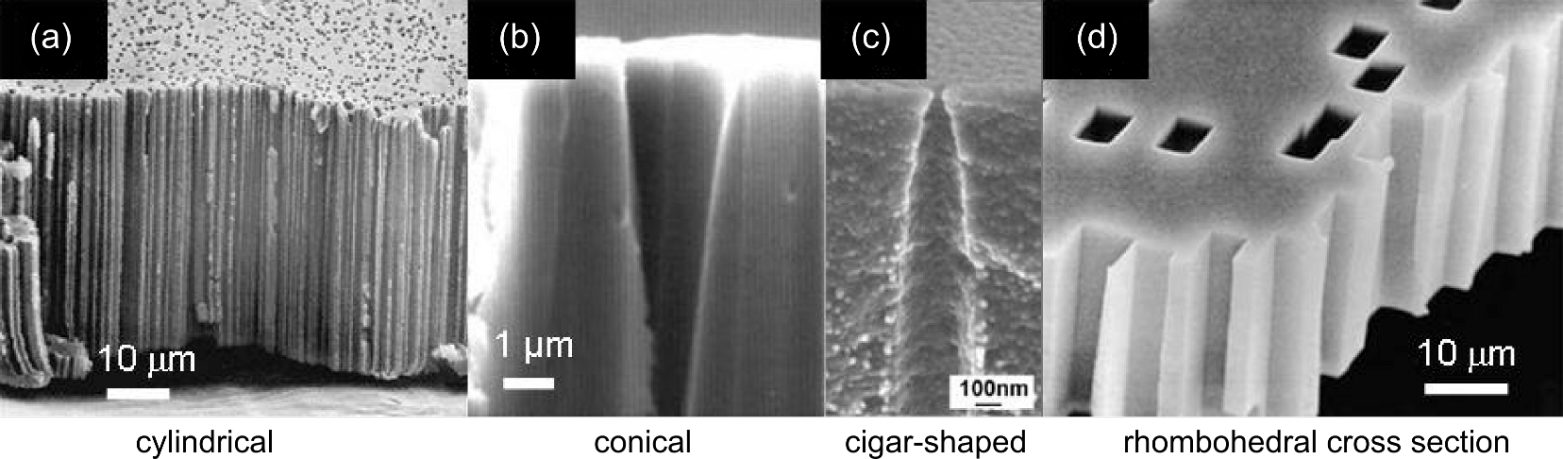
SEM images displaying the cross sections of the following membranes: (a) cylindrical channels in PI; (b) conical channels in PI; (c) cigar-shaped channels in PET, (d) cylindrical channels with rhombohedral cross section in mica. (a,b) Adapted with permission from [26] – Copyright 1996 Elsevier; (c) adapted with permission from [42] – Copyright 2007 IOP Publishing Ltd; (d) adapted with permission from [43] – Copyright 2012 American Physical Society.

**1.1.2. Chemical etching:** In a suitable etching solution the ion tracks can be selectively dissolved and subsequently enlarged into channels [39]. For the successful fabrication of templates, the anisotropic dissolution rate along the ion track (*V*_t_) must be higher than the dissolution rate of the undamaged bulk material (*V*_b_). The material of choice together with the etching conditions (temperature, composition, and concentration of the etchant) determine the track-to-bulk etching ratio (*V*_t_/*V*_b_) and thus also the geometry of the channels. High *V*_t_/*V*_b_ ratios result in the formation of cylindrical channels (Figure 3a and Figure 3d), whereas low ratios result in conical (Figure 3b) or biconical channels. High etching selectivity is achieved in PI by using, e.g., sodium hypochlorite (NaOCl) [26], while tracks in PET and PC are preferentially etched in sodium hydroxide (NaOH) solutions [34,39]. Exposure of ion-irradiated polymers to UV light prior to etching increases *V*_t_ and leads to a narrower size distribution of the channels [40–41].

**Figure 4:**
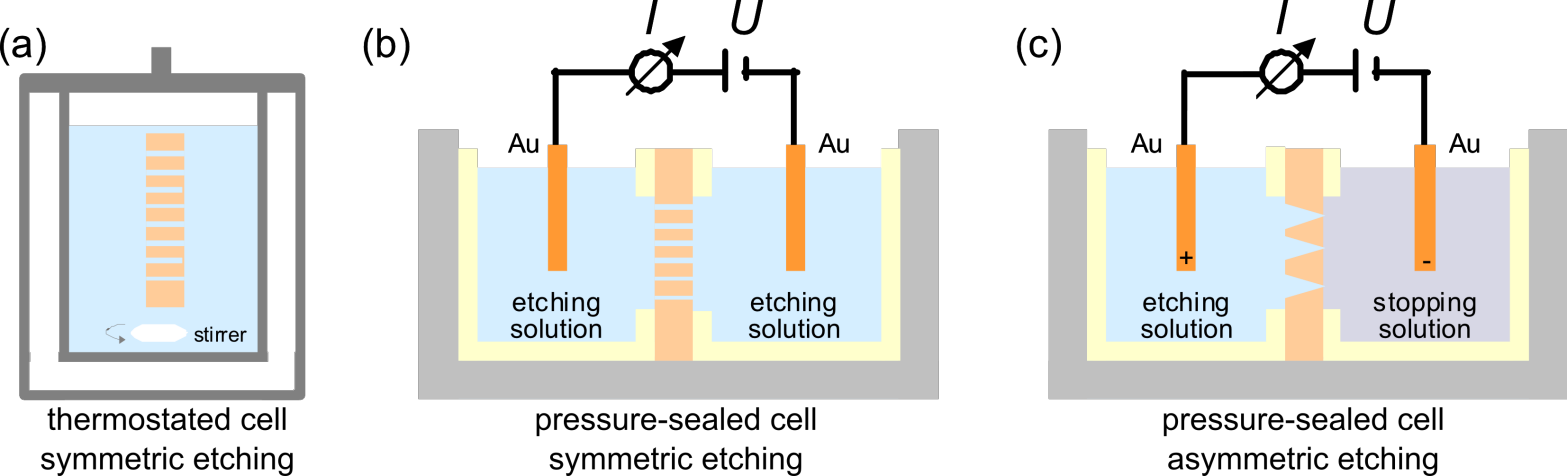
Schematic of etching equipment. Symmetric etching conditions leading to cylindrical channels in (a) thermostated cell and (b) pressure-sealed cell. (c) Asymmetric conditions leading to conical channels in pressure-sealed cell. Electric current measurements allow monitoring of the etching process.

Symmetric etching of cylindrical channels is performed in a thermostated etching bath (Figure 4a). Stirring improves convection and provides a homogeneous temperature of the bath. Alternatively, chemical etching in both symmetric and asymmetric configuration can be performed in a two-compartment electrolytical cell at constant temperature (Figure 4b and Figure 4c). The irradiated foil is sealed between the two half-cells. Exposing the foil surfaces to different solutions (e.g., etching and neutralizing agents), allows one to adjust different conditions. By means of current measurements, the etching process is monitored online. A voltage *U* is applied between two gold electrodes immersed at each side of the foil and the current *I* is recorded with a picoammeter as a function of time. While the pore has not yet been etched through, the membrane acts as a very large resistance, and no current flows. As soon as the track is converted into an open channel, the electric current *I* starts to increase. The etching is continued and the current will increase as the pore diameter enlarges [31].

For the fabrication of cylindrical multi- or single pores in PC, the etching process is performed in a symmetric configuration by exposing the foil to concentrated NaOH solution on both membrane sides (Figure 4b) [34,44–45]. To obtain conical nanopores, one half-cell is filled with a suitable etchant while the other half-cell contains either water or an acidic stopping medium that neutralizes the etchant as soon as the pore opens. In both cases, further etching is extensively slowed down or entirely stopped (Figure 4c). In addition, by immersing the positive anode in the etching solution, the negative ions in the etchant migrate away from the pore tip when the pore breaks through. This helps to create channels of reduced tip diameter [46].

Conical nanopores in PET, PC, and PI have been produced with different combinations of etching and stopping solutions. For example, conical channel geometries in PC and PET are typically achieved by using solutions of sodium hydroxide (various concentrations) for etching and a mixture of potassium chloride (KCl) and formic acid (HCOOH) for stopping [31,46]. Methanol can be added in different concentrations to the NaOH etchant to influence *V*_b_. In the case of 30 µm thick PC foils, it was reported that with an increasing volume concentration of methanol from 0 to 80%, the cone half-angle increases from about 0.2 to about 3.6° [47]. In the case of PI, the etching is typically performed in sodium hypochlorite (NaOCl) solution with an initial pH value 12.6 and an active chlorine content of 13%, while KI acts as reducing agent for the OCl^−^ ions of the etchant [32,48]. The apex angle of the conical pores in PI becomes larger by increasing the pH of the NaOCl solution [26]. Channels with specific geometries other than cylindrical or conical, for example, cigar-shaped (Figure 3c), are fabricated by applying adequate surfactants [46,49]. Before further processing, the template is rinsed in purified water.

Compared to other available templates, such as di-block copolymer membranes or porous alumina, etched ion-track membranes offer the powerful possibility of controlling all important parameters of the synthesized nanostructures in an independent manner: (i) The irradiation fluence determines the preset density of parallelly oriented nanochannels; (ii) By tilting the samples in the ion beam, tilted channels can be produced yielding an interconnected nanochannel network; (iii) The polymer material of choice, together with the etching conditions, determine the geometry of the channels, e.g., cylindrical, conical, and biconical; (iv) Controlled by the etching time, uniform channels can be produced with diameters from about 10 nm to a few micrometres.

#### 1.2 Electrodeposition of nanowires

This section presents the setting for nanowire electrodeposition, and discusses the electrochemical deposition processes as analysed by chronoamperometric monitoring.

**Figure 5:**
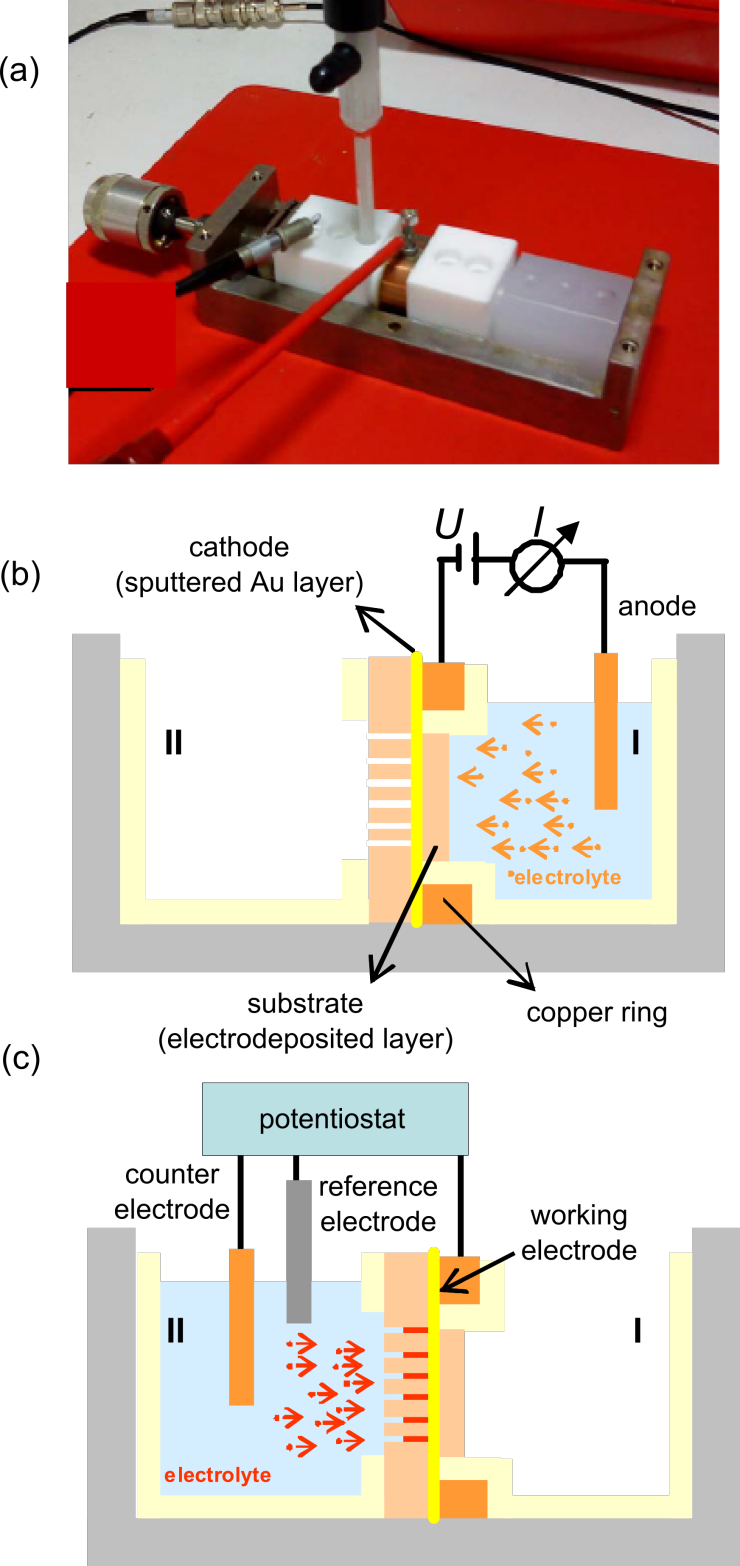
(a) Photograph of a pressure-sealed electrochemical cell. (b) Schematic for the electrodeposition of a conductive layer as substrate in a two-electrode configuration (cathode and anode). (c) Schematic for the growth of nanowire arrays by using three electrodes (working, counter, and reference electrode).

**1.2.1 Electrochemical cells:** The photograph in Figure 5a shows our electrochemical cell, currently in use. The polymer foil is placed between two polytetrafluorethylene compartments, and is sealed by mechanical pressure. A good sealing is essential to avoid leak currents, an important requirement when growing single nanowires. Very small currents in the picoamp range must be recorded and analysed, as demonstrated during electrodeposition of nanowires, e.g., Cu and Bi, in single-nanopore membranes [35–36,50–51]. This pressure-sealed cell is also suitable for the growth of nanowire arrays of various materials [52–57]. Figure 5b and Figure 5c depict schematically the two deposition processes involved in the nanowire synthesis: substrate deposition and nanowire growth, respectively. First, one side of the track-etched polymer membrane is sputter coated with a thin (few ten nanometres) Au layer. The membrane is then mounted between the two cell compartments. The sputtered metal layer is in contact with a copper ring accessible to external electronic equipment (voltage supply or potentiostat). In some cases, the thin conductive Au layer is reinforced electrochemically by a metal layer (e.g., Cu, Au) in a two electrode configuration (Figure 5b). After reinforcement, the electrolyte is removed from the first compartment (I) and the membrane is rinsed with distilled water. For the electrochemical deposition process, the specific electrolyte is introduced in the second compartment (II) and an adequate deposition potential is applied. At a preselected constant temperature, the nanowires then grow from the bottom-side (sputter-coated layer) to the top-side of the membrane (Figure 5c).

The wire synthesis can be performed by using a two- or a three-electrode arrangement, under potentiostatic or galvanostatic conditions. In the case of potentiostatic and pulsed deposition, the process is monitored by chronoamperometric current–time (*I*–*t*) curves. In the two-electrode arrangement the potential *U*_c_ is applied between cathode and anode. In the three-electrode arrangement, reference electrodes such as saturated silver/silver chloride (Ag/AgCl/sat. KCl) and saturated calomel electrodes (SCE) are currently employed. Larger thermostated cells have also been employed, e.g., for the growth of Cu and Bi_2_Te_3_ nanowires, to improve convection by magnetic stirring or to provide temperatures above or below ambient conditions [58–59].

**Figure 6:**
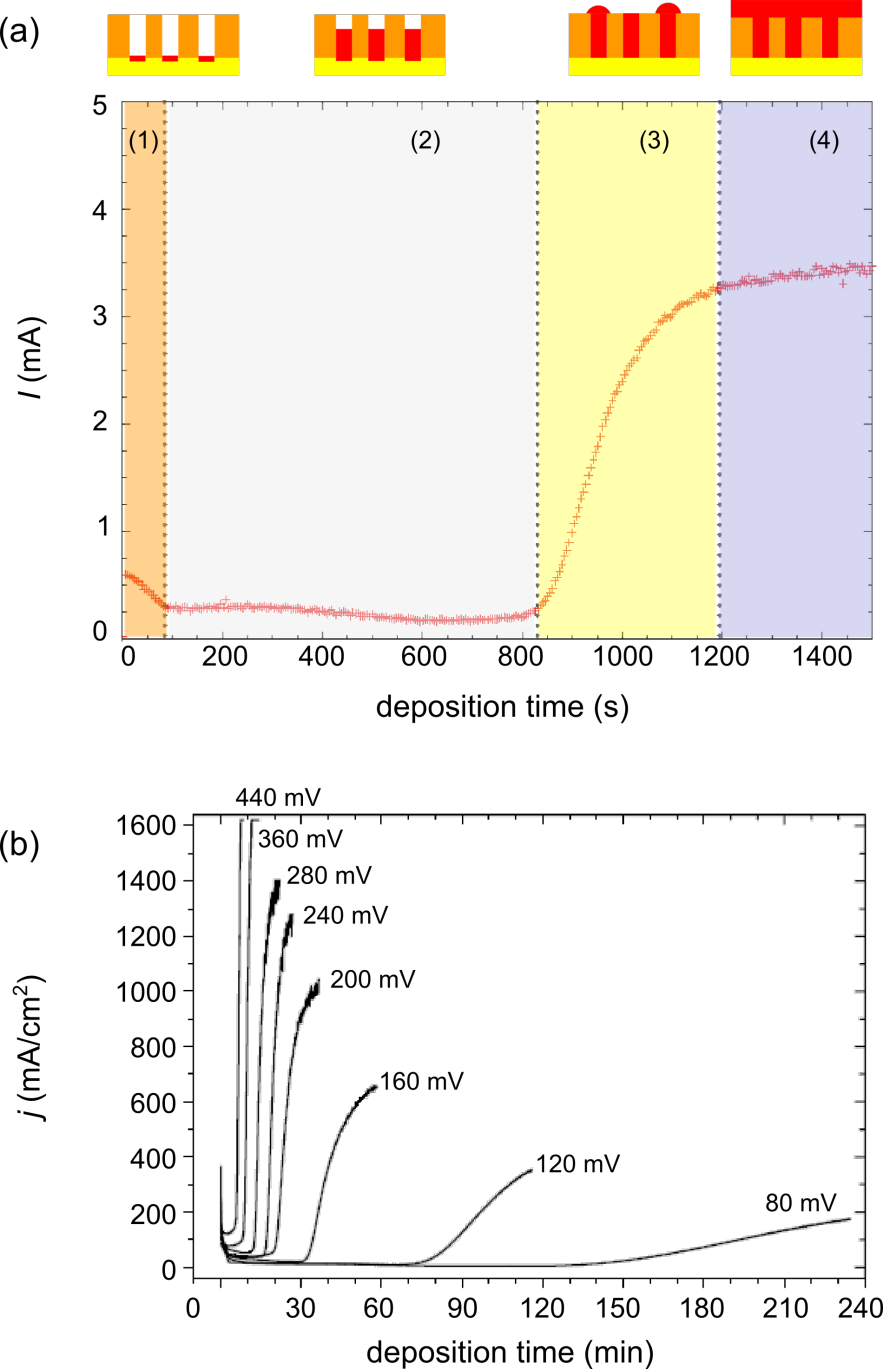
(a) Representative *I*–*t* curve and schematic of the four different deposition regimes. (b) Chronoamperometric curves recorded during growth of Cu nanowires in identical templates (30 µm thick PC, 10^7^ channels/cm^2^, pore diameter 450 nm) in a solution consisting of 0.25 mol/L CuSO_4_·5H_2_O and 2 mol/L H_2_SO_4_, by applying different potentials (versus Ag/AgCl/3 mol/L KCl provided with a Haber–Luggin capillary). Adapted with permission from [59] – Copyright 2003 The Electrochemical Society, Inc.

**1.2.2 Chronoamperometric monitoring:** During the potentiostatic growth of nanowires, four different current regimes can be identified (Figure 6a): (1) A sharp decrease of the current at the beginning of the process attributed to the creation of the diffusion layer; (2) nanowire growth inside the channels with nearly constant current; (3) more or less sharp current increase when the material reaches the top side of the membrane and caps start to grow on top; and (4) if the process is continued, the caps grow further and eventually form a continuous layer. Current–time characteristics displaying these four distinct regions have been reported for the growth of Cu [52], Au [53], Bi [56], Pt [55], Bi_1−_*_x_*Sb*_x_* [60], Bi_2_Te_3_ [58], and Ni nanowires among others. The integral of the *I*–*t* curves between the beginning of the deposition and the transition to zone 3 corresponds to the charge *Q*_exp_ applied during the growth process. Assuming complete pore filling, the expected total charge *Q*_theo_ is given by the Faraday law, namely *Q*_theo_ = (*z*·*F*·*m)/M*, with *z* being the number of electrons transferred per ion during the reaction, *F* the Faraday constant (96.485 C·mol^−1^), *m* the total mass and *M* the molar mass of the deposited substance. In the case of 100% efficient electrochemical reactions, the ratio *Q*_exp_/*Q*_theo_ is an indicator of the homogeneity of the wire growth over the whole sample. *Q*_exp_ < *Q*_theo_ indicates that deposition has not occurred in all channels simultaneously, and/or that the number density of the wires is lower than that of the channels. Monitoring the *I*–*t* curves during homogeneous growth allows us to stop the deposition after a given time to obtain nanowires of a predefined length. By this technique, wires of length between 1 and 60 µm were fabricated. Figure 6b shows representative chronoamperometric curves recorded during the potentiostatic growth of Cu nanowires in PC membranes (*d*_pore_ = 450 nm, 10^7^ cm^−2^) at 25 °C, in a solution consisting of 0.25 mol/L CuSO_4_·5H_2_O and 2 mol/L H_2_SO_4_, by applying different potentials ranging between −80 and −440 mV (versus Ag/AgCl/3 mol/L KCl provided with a Haber–Luggin capillary) [59].

### 2 Composition and crystallinity of the nanowires

During electrochemical growth of nanowires, two mechanisms occur simultaneously inside the membrane channels: (i) nucleation of new grains and (ii) growth of existing nuclei. To synthesize single-crystalline micro- or nanowires, the fabrication conditions should be chosen such that the second process dominates [61]. It should be noted, that other than on a macroelectrode surface, the wire deposition process occurs in a recessed electrode ensemble, and that the cathode surface is placed, at the beginning of the process, at the bottom of the channels and shifts to the opposite surface during the nanowire growth [59].

Control over the crystallinity is especially important when the size of the investigated nanostructures is comparable to characteristic length scales such as electron and phonon mean free paths and Fermi wavelength. For nanomaterials, phenomena such as electrical and thermal resistivity or magnetoresistance are known to depend strongly on their crystallinity and morphology [62–64]. Also the surface plasmon resonances show pronounced effects on size, material, and shape [65–66]. A detailed morphological and crystallographic characterization of the synthesized nanostructures is required (i) to understand how the synthesis parameters influence the resulting crystalline structure, and (ii) to investigate size-dependent nanowire properties relevant for different applications. The following sections present results from systematic studies of the influence of the growth parameters on the resulting crystallinity and morphology of nanowires of various materials.

#### 2.1 Copper nanowires

Copper is an important material for the microelectronic industry due to its low resistivity and its low vulnerability to electromigration, a phenomenon that produces voids in wires and ultimately causes failure. Copper micro- and nanostructures in addition are synthesized for applications in solar cells, flat-panel displays, and sensorics. The most common approaches to synthesize copper micro- and nanostructures include electrodeposition, chemical vapour deposition, electroless deposition, and solution growth [67–69]. Among them, electrochemical deposition is most suitable for fabrication of nanostructures in trenches of small dimensions and/or high aspect ratios (length/diameter) [70].

**Figure 7:**
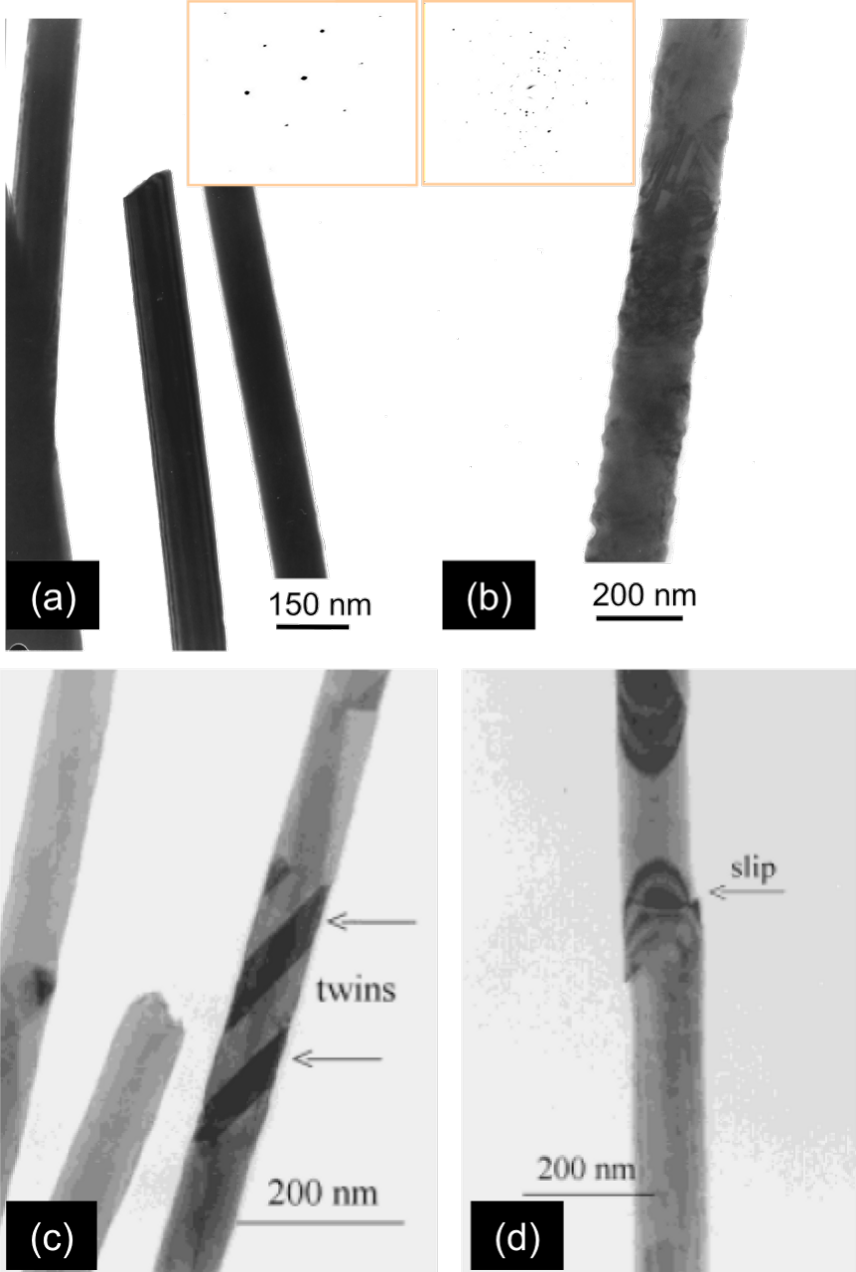
TEM images of representative Cu nanowires and lattice defects: (a) single-crystalline and (b) polycrystalline Cu wires together with their corresponding SAED patterns (insets). (c) 70 nm diameter single-crystalline wire with twin structures. (d) Slip in a 100 nm diameter Cu wire. (a,b) Adapted with permission from [71] – Copyright 2001 Elsevier and (c,d) adapted with permission from [52] – Copyright 2001 Wiley-VCH.

Based on the above-described template technique, poly- and single-crystalline Cu nanowires with aspect ratios above 500 and diameters as small as 30 nm were synthesized by electrodeposition in PC etched ion-track membranes. A suitable electrolyte is, e.g., an aqueous solution containing 238 g/L CuSO_4_·5H_2_O and 21 g/L H_2_SO_4_ [52]. The high concentration of CuSO_4_ guarantees a sufficiently large supply of ions inside the pores during the deposition. Addition of sulphuric acid increases the conductivity of the solution and lowers the cathode overvoltage. Electrodeposition is typically performed potentiostatically in a two-electrode arrangement by using a copper anode, at temperatures between 25 and 70 °C. By applying low overvoltages, side reactions, such as hydrogen evolution, are avoided. Figure 7 displays transmission electron microscopy (TEM) images of representative (a) single- and (b) polycrystalline Cu nanowires together with their respective selected-area electron diffraction (SAED) pattern. The single-crystalline Cu wire was deposited at 50 °C by applying a voltage of *U*_c_ = −50 mV. The wire exhibits cylindrical geometry with constant diameter and a smooth contour over the entire length. The polycrystalline wire was deposited at room temperature and at a larger negative potential. Its contour is clearly rougher, probably due to the higher growth rate. Increase of surface roughness with increasing deposition potential has also been observed in the case of Bi compound wires [58]. X-ray diffraction (XRD) performed on the single-crystalline nanowire arrays by using a four-circle diffractometer, revealed a preferred orientation of the (110) planes perpendicular to the wire axis [71].

Recently, Duan et al. presented an exhaustive investigation of the preferred orientation of Cu nanowire arrays, demonstrating that their preferred crystallographic orientation can be adjusted along the [111], [100] or [110] directions by selecting specific parameters with respect to the sulphuric acid concentration in the electrolyte, the applied voltage, and the deposition temperature [72]. It was also reported that single-crystalline Cu microwires were grown under room-temperature conditions by using commercial baths and reverse-pulse plating in an ultrasonic bath in a two-electrode arrangement [73]. Copper nanowires were also synthesized in a three-electrode arrangement by using a SCE as reference electrode [59]. Cylindrical multilayered Cu/Cu_2_O nanowires were electrochemically deposited from the self-oscillating Cu(II)-lactate system by using PC templates [74].

Figure 7c shows the TEM image of a twinned region, as frequently found in single-crystalline Cu wires. Twinning is a crystal defect characterized by the partial displacement relative to the matrix of a considerable number of neighbouring crystallographic planes [75–76] and is evident by the reduced brightness in Figure 7c. Twins can be created during the growth process but may also result from plastic deformation when handling the samples. Also slips are frequently observed, not only in Cu wires (Figure 7d) but also in other materials such as Au and Bi_2_Te_3_. Planar defects such as twinning or slips are expected to influence the electrical and thermal transport properties, as well as the mechanical stability of nanowires.

#### 2.2 Gold nanowires

Numerous theoretical predictions and experiments have demonstrated that Au nanoparticles and nanowires are promising elements for sensoric, optical and biomedical applications. Of special interest are surface plasmon resonances (SPRs) of Au nanostructures, because electromagnetic radiation is confined to a volume of sub-wavelength dimensions. It is known that field enhancements due to SPRs are strongly dependent on size, geometry, and composition of the nanostructures [65–66].

Systematic studies were performed on the electrochemical template synthesis of Au-nanowires in a two-electrode configuration by using a sputtered Au film as initial cathode and a Au rod as anode. The investigations provided adequate growth conditions for both single- and polycrystalline wires with diameters between 20 and 100 nm [53–54]. Other than for copper, nanowires deposited by using the ammonium gold(I) sulfite electrolyte (gold content = 15 g/L, Metakem GmbH, Usingen, Germany), or the sodium disulfitoaurate(I) Imabrite 24 bath (gold content = 12*.*3 g/L, Schloetter Galvanotechnik, Geislingen/Steige, Germany) exhibit a polycrystalline structure independently of temperature and voltage. In contrast, wires grown in a solution of potassium dicyanoaurate(I) (Puramet 402 bath, gold content = 10 g/L, Doduco, Pforzheim, Germany) yield single crystals at temperatures between 50 and 65 °C under both direct-current and reverse-pulse deposition conditions. The resulting single-crystalline wires have a preferred [110] orientation.

**Figure 8:**
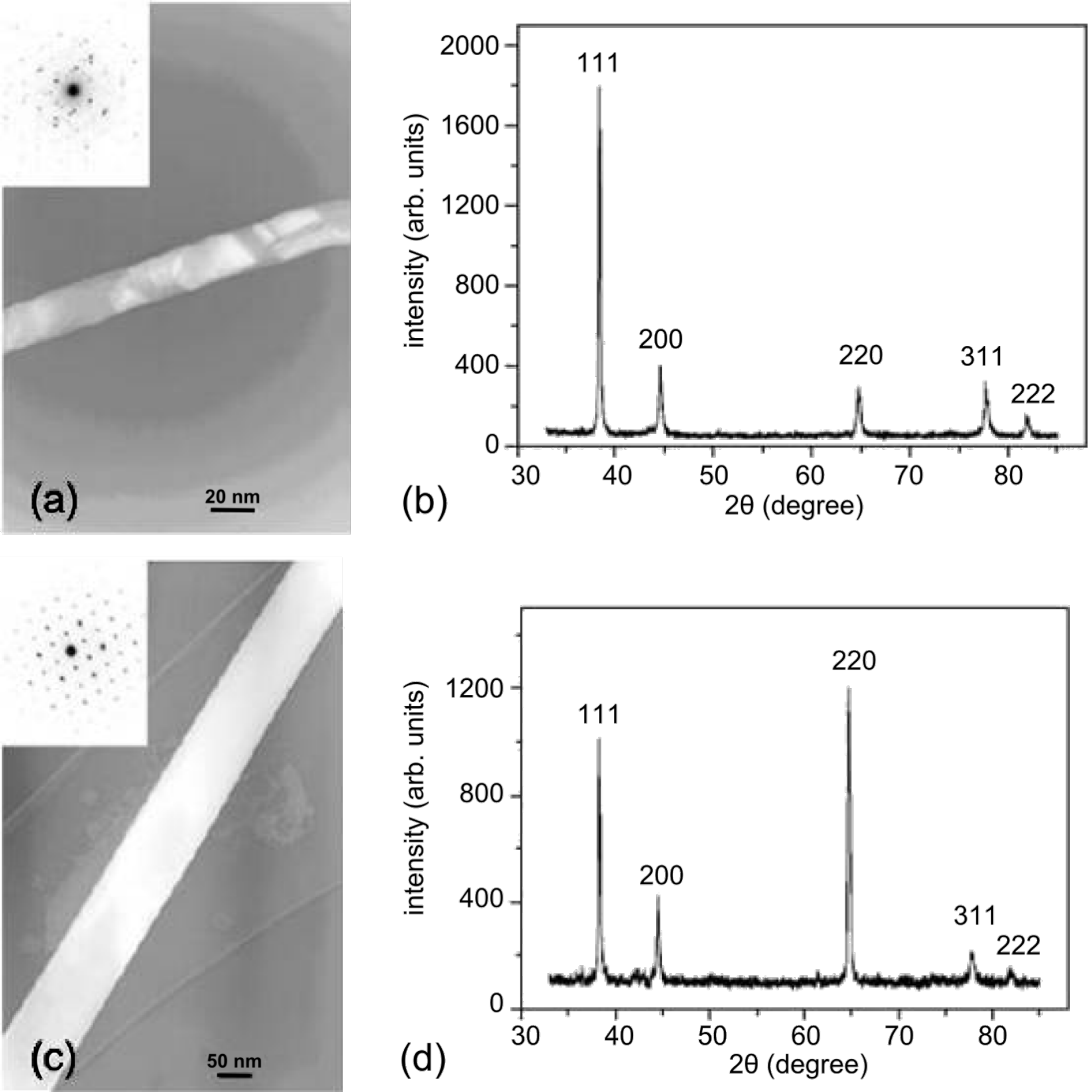
TEM images of representative (a) polycrystalline and (c) single-crystalline Au nanowires and (b,d) their corresponding XRD diffractograms measured on nanowire arrays deposited under the same conditions. Adapted with permission from [53] – Copyright 2006 IOP Publishing Ltd.

Figure 8a shows a representative TEM image of a polycrystalline Au nanowire deposited with an ammonium gold(I) sulfite electrolyte, at 50 °C, by applying *U*_c_ = −500 mV between cathode and anode. Several zones of light and dark contrast reveal several grain boundaries along the wire axis. The TEM image in Figure 8c depicts a single-crystalline wire deposited with the cyanidic electrolyte at *U*_c_ = −900 mV and 60 °C, while the cell was immersed in an ultrasonic bath. The authors reported that the presence of ultrasound fields improved the convection in the pores and thus the homogeneity of the growth on the whole sample, leading to homogeneous wire arrays. The crystallinity of the different wires is confirmed by the respective SAED patterns (insets). Further, the XRD pattern of the polycrystalline wires indicates random orientation (Figure 8b), while single-crystalline wires have a preferred orientation of the (110) planes perpendicular to the wire axis (Figure 8d).

#### 2.3 Nanowires from other metals

In the recent past, nanowires of a number of different metals have been synthesized, including Pt, Pb, Ni, Co, and Fe. Platinum is a noble metal with interesting nanowire applications in sensorics and catalysis.

Recently, the synthesis of Pt nanowires at 65 °C from an alkaline platinum bath (Platinum-OH, Metakem) in a two electrode arrangement by using a Pt rod as anode was reported by Rauber et al. [55]. Contrary to copper and gold, the crystallinity of nanowires of metals with high melting temperatures, such as Pt and Rh, is difficult to control through the deposition parameters (i.e., to control nucleation and surface diffusion processes at the cathode). TEM and XRD investigations revealed a fine-grained polycrystalline structure for all potentiostatic conditions applied. Multisegmented polycrystalline Pt nanowires with preset and controlled number of segments/interfaces were synthesized by pulse-reverse electrodeposition. The cathodic pulse was applied at a potential *U*_c_ = −1.3 V for different pulse durations ranging from *t*_c_ = 1 s to *t*_c_ = 20 s. The anodic pulse was invariably timed to *t*_a_ = 1 s at *U*_a_ = 0.4 V. In this process, the length of the segments is controlled by the duration of the cathodic pulse [55].

As demonstrated by Yi and Schwarzacher, single- and polycrystalline Pb nanowires of 50 nm diameter grow reproducibly in etched ion-track membranes for various pulse parameters. An interesting finding is that the superconducting transition temperature *T*_c_ depends on the crystallinity of the nanowires [78].

Magnetic nanowires were successfully grown from nickel [57], cobalt [79], and iron [80]. The growth of Fe-based nanowires with controllable size, aspect ratio, and magnetic anisotropy in FeCl_3_ and FeCl_2_ solutions was investigated by Song et al. They employed FeCl_3_ and FeCl_2_ solutions, studied the nanowire growth mechanism and provided real-time compositional and crystallographic information [80].

#### 2.4 Bismuth and bismuth-compound nanowires

Due to its unique electronic properties, bismuth is a very interesting material to study the effect of finite- and quantum-size effects of nanostructures [9,81–82]. Characteristic length scales, such as the electron mean free path and Fermi wavelength are relatively large at room temperature, namely 100 and 40 nm, respectively [83–84]. Bulk Bi is a semimetal with a very small indirect band overlap, and its charge carrier density is low compared to conventional metals. Moreover, the electron effective mass is small (0.001–0.26) and depends on the crystalline orientation. Given these characteristics, size effects on Bi structures are expected at relatively large dimensions (≈100 nm). Also compound nanostructures of Bi_1−_*_x_*Sb*_x_* and Bi_2_Te_3_ are being intensively investigated due to theoretical studies predicting a large enhancement of the thermoelectric efficiency, given by the so-called figure of merit *ZT*, *ZT* = *S*^2^·σ·*T*/κ, where *S* is the Seebeck coefficient, σ is the electrical conductivity, κ is the thermal conductivity and *T* is the temperature. The power factor (*S*^2^σ) of these thermoelectric nanomaterials should increase due to quantum size effects and the thermal conductivity should decrease due to enhanced phonon surface scattering [85–88]. The thermoelectric properties of these Bi-compound materials are anisotropic and are extremely sensitive not only to composition and size, but also to the crystallographic orientation of the wires. During synthesis, it is thus important to control these three parameters simultaneously. To achieve a significant enlargement of the thermoelectric efficiency, the diameter of such nanowires should be below 30 nm.

**Figure 9:**
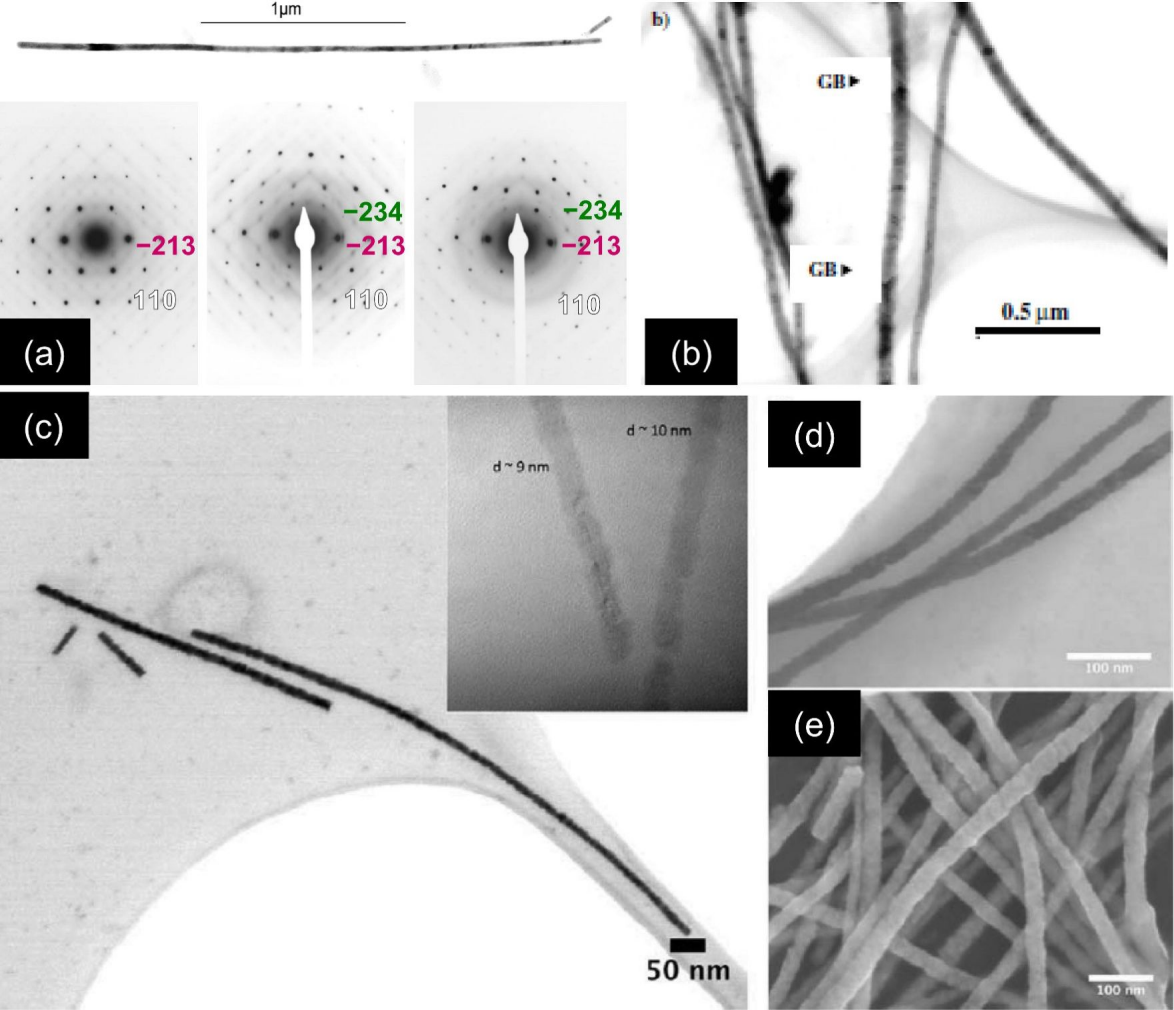
TEM images of Bi and Bi_2_Te_3_ nanowires: (a) individual single-crystalline Bi nanowire deposited under potentiostatic conditions together with SAED patterns from different wire positions and (b) several polycrystalline wires grown under pulsed conditions. Series of SEM images displaying Bi_2_Te_3_ nanowires with average diameters of (c) 14, (d) 19 and (e) 24 nm. The inset displays TEM images of sections with diameter 9–10 nm. (a,b) Adapted with permission from [56] – Copyright 2005 IOP Publishing Ltd and (c–e) adapted with permission from [58] – Copyright 2012 American Chemical Society.

Cornelius et al. fabricated pure Bi nanowires using an electrolyte consisting of 0.2 M BiCl_3_, 0.3 M tartaric acid, 0.2 M NaCl, 1.3 M HCl, and 100 g/L glycerol, in most cases potentiostatically, but also using reverse-pulse deposition in a two-electrode arrangement [56]. The thin Au layer acted as cathode and a Bi rod as anode. XRD and TEM revealed that the nanowires deposited potentiostatically are <110> textured. At higher temperatures and smaller overpotentials, the texture increases. At *T* = 60 °C and low overpotentials (e.g., *U*_c_ = −17 mV), single-crystalline wires are produced (Figure 9a). In contrast, wires deposited with reverse pulses exhibit a <100> texture and are polycrystalline with grain sizes of ca. 0.5 µm (Figure 9b). Bi nanowires with other preferred orientations have been synthesized by other techniques, such as low-temperature solvothermal process and high-pressure injection in alumina [89–90].

Recently, Bi_2_Te_3_ nanowires with diameters from 150 nm down to 10 nm, and lengths of up to 60 µm, were potentiostatically grown by using a thermostated three-electrode setup with a thin sputtered Au layer acting as the cathode, a Pt counter electrode, and a SCE as the reference electrode [58]. The electrolyte consisted of an aqueous solution of bismuth nitrate pentahydrate, TeO_2_, and nitric acid. As shown by means of XRD, TEM, SEM, and EDX (energy-dispersive X-ray analysis), the parameters involved in the electrodeposition process, *T*, *U*, and diameter, density, and length of the channels in the template, influence the morphology, crystallinity, and preferred crystallographic orientation of the wires in a complex manner. The Bi_2_Te_3_ nanowires have diameters and lengths interesting for both basic research on thermoelectric nanomaterials and development of thermoelectric devices. Figures 9c–e display SEM images of Bi_2_Te_3_ nanowires with average diameters (c) 14, (d) 19 and (e) 24 nm. The smallest Bi-compound wires synthesized so far had diameters as small as 9–10 nm (inset). To the best of our knowledge, with 14 nm average diameter, 10 µm length, and aspect ratios between 700 and 1000, these are presently the thinnest nanowires produced by electrodeposition in polymer membranes.

**Figure 10:**
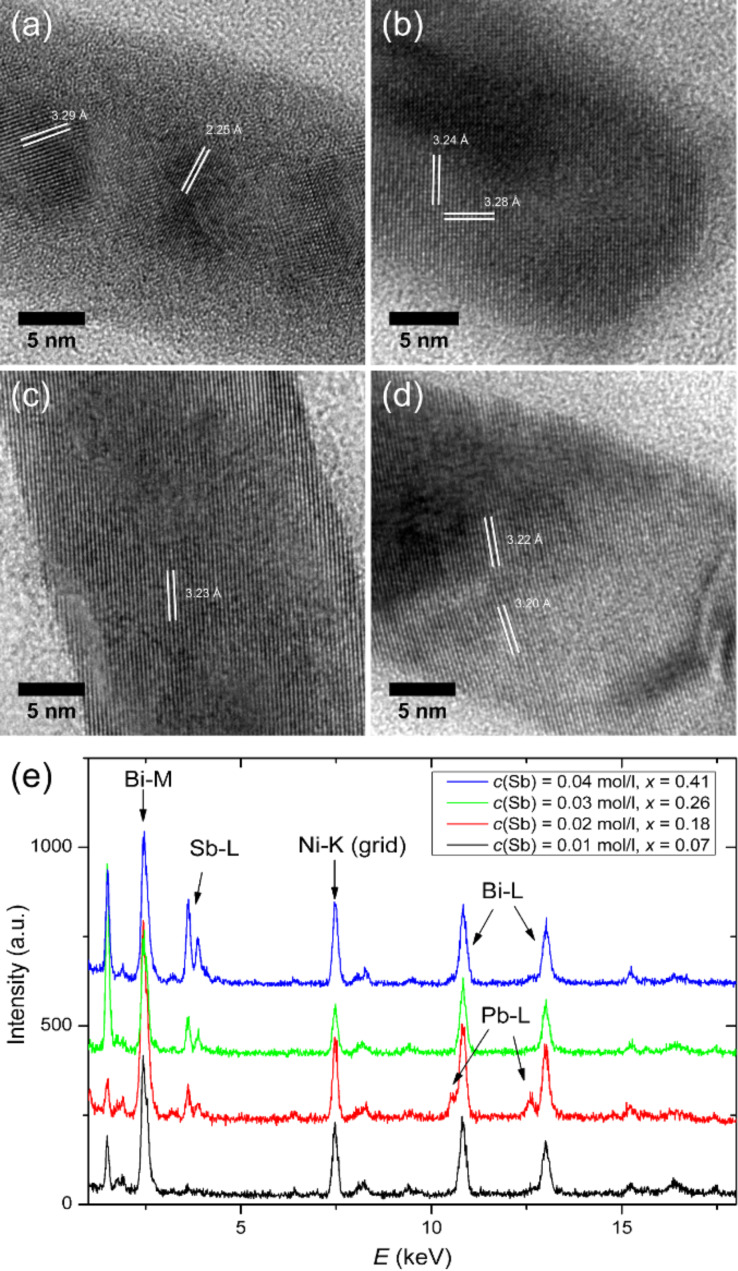
TEM images of Bi_1−_*_x_*Sb*_x_* nanowires deposited at *U* = −200 mV versus SCE from electrolytes with Sb concentrations (*c*(Sb)) of (a) 0.01, (b) 0.02, (c) 0.03 and (d) 0.04 mol/L. (e) Corresponding EDX spectra, indicating the following Sb concentration in the wires x: 0.07, 0.18, 0.26 and 0.41. Adapted with permission from [60] – Copyright 2011 American Chemical Society.

Polycrystalline Bi_1−_*_x_*Sb*_x_* nanowires were successfully electrodeposited from an aqueous solution of BiCl_3_ and SbCl_3_, with simultaneous control over the diameter (between 20 and 200 nm), and varying Sb concentration (0.05 ≤ x ≤ 0.4). Coarse- and fine-tuning of the Sb concentration was achieved by selecting proper electrolyte composition and potential [60]. Figure 10 displays HRTEM images of 20−30 nm diameter nanowires deposited at *U* = −200 mV versus SCE and for different Sb concentrations in the electrolyte (*c*(Sb) = 0.01 (a), 0.02 (b), 0.03 (c), and 0.04 mol/L (d)), together with their respective EDX spectra (e). On average, the concentration of Sb in the wires was found to be *x* = 0.07, 0.18, 0.26, and 0.41, respectively. With increasing Sb concentration, the *d*-spacings belonging to the {012} lattice planes decrease, as evident by white lines in the HRTEM images (Figures 10a–d). XRD investigations on the preferred crystallographic orientation of Bi_2_Te_3_ and Bi_1−_*_x_*Sb*_x_* nanowires grown in templates are described in references [58,60].

All experimental results reported so far clearly demonstrate that electrodeposition of thermoelectric nanowires in etched ion-track membranes enable the control of various wire parameters. It is particularly important to control the alloy composition and to obtain wire diameters as small as possible, because large enhancements in TE performance are expected when quantum size effects and enhanced phonon scattering come into play.

#### 2.5 Semiconductor nanowires

Semiconductor nanowires are excellent candidates to be functional elements in applications as diverse as optics, sensorics, and electronics, and energy applications such as thermoelectrics and hydrogen generation by water splitting [2–4,91]. In the past two decades, enormous progress has been achieved in the synthesizing and characterizing of semiconductor nanowires of controlled size and composition. Synthesis techniques include mostly vapour–liquid–solid growth, solution-phase, lithography, and electroless etching [2,10], while the template method in combination with electrodeposition of semiconductor nanowires (such as ZnO, Si, or ZnTe) has been rather limited.

Cylindrical ZnO nanowires have been electrochemically grown from aqueous solutions in the pores of both alumina and etched ion-track membranes with a rather limited range of diameters. Lai et al. reported the synthesis of ZnO nanowires using a ZnSO_4_-based electrolyte at 22 °C, and a Zn(NO_3_)_2_-based solution at 70 °C [92]. Enculescu and co-authors reported the fabrication and optical characterization of ZnO wires with diameters between 80 nm and 1.5 µm, deposited in etched ion track membranes using a Zn(NO_3_)_2_-based electrolyte at 70 °C, with a Pt foil and a SCE electrode as counter and reference electrodes, respectively [93]. By appropriately tuning the composition of the electrolyte, they also synthesised doped ZnO nanowires. By using, for instance, an electrolyte containing Zn(NO_3_), Co(NO_3_), nitric acid, and polyvinylpyrrolidone (PVP) as an additive, 300 nm diameter Zn_1−_*_x_*Co*_x_*O nanowires with *x* ranging from 0.01 to 0.05 were grown [94].

The synthesis and properties of semiconducting CdTe and CdS nanowires are being investigated for their potential in photodetector and photovoltaic applications. CdTe and CdS rods are mostly synthesized by chemical vapour deposition, and sol–gel processes. Electrodeposition of stoichiometric CdTe nanowires with diameters between 80 nm and 1 μm was reported by Enculescu et al. [95]. In addition to SEM, TEM, EDX, and XRD characterization, they also determined the band gap of nanowire arrays by reflection spectroscopy measurements [96]. Kum et al. reported the synthesis of ≈50 nm diameter CdTe wires and studied the influence of electrolyte, temperature, potential, and pH value on the composition and crystallinity of the nanowires [97]. They also demonstrated that as-deposited CdTe nanowires consist of nanocrystals with grain sizes up to 60 nm. Thermal annealing increases the wire resistivity and influences the grain size. The preparation of CdTe nanowire diodes with semiconductor homojunctions by using a single electrodeposition bath consisting of cadmium sulfate (0.02 M) and tellurium dioxide (1 mM) as sources of cadmium and tellurium ions, respectively, was described by Matei et al. [98]. The tellurium dioxide was dissolved in 50% concentrated sulfuric acid and the overall pH was adjusted to 2 with sodium hydroxide. Polyvinylpyrrolidone (1 g/L) was added as a wetting agent. A Pt foil and a SCE acted as counter and reference electrode, respectively. The potentiostatic electrodeposition of CdS nanowires by using an electrolyte solution containing CdCl_2_ and thioacetamide, at 70 °C was reported by Mo et al. [99].

Finally, due to its availability, inertness, and compatibility with silicon-based technical processing, Si nanowires have a broad range of applications from sensorics, to biotechnology, photonics, IR-sensorics, and many others. Si wires have been prepared by a large variety of deposition techniques, including chemical vapour deposition, laser ablation, or thermal evaporation. The first template-grown nanowires of amorphous Si were recently reported by using ionic liquids [100–101]. Ionic liquids have proved to be a good alternative electrolyte to fabricate materials such as Al, Ti, Si, or Ge, which cannot be electrodeposited in aqueous solvents [102]. Given the extreme versatility of etched ion-track membranes, future electrochemical growth of Si nanowires would allow tuning size parameters and provide interesting freestanding high-aspect ratio Si nano- or microstructures [103].

#### 2.6 Segmented nanowires

Besides synthesizing single metal and semiconductor nanowires, electrodeposition also offers the possibility to grow segmented multimaterial nanowires. Combining various materials of interest can provide specific functionalities that are not present in the individual segments. Variations in composition along the length of the wire can, e.g., be used to incorporate electrical functionality, optical contrast, and/or desired surface chemistry [104]. Segmented Au/Pt nanowires were demonstrated to move autonomously when placed in a hydrogen peroxide solution [105]. Also, biofunctionalized nanowire bar codes were used for ss-DNA detection [106]. In addition, it is also of interest to grow metal segments on both sides of a semiconductor nanowire in order to provide electrical contacts.

To synthesize two-component multisegment nanowires, a single electrolyte bath containing the two ions of interest is employed. At less negative potentials, only the more noble metal is deposited, while at more negative potential, both metals are deposited. By keeping the concentration of the more noble metal in the electrolyte much lower than the concentration of the less noble metal, the less noble metal is mainly deposited containing a small fraction of the nobler one [64]. Alternatively, a two-bath sequential deposition can be employed. Multilayer nanowires were electrodeposited in etched ion-track membranes in the nineties to study the perpendicular-to-plane giant magnetoresistance (GMR) [64]. Multilayered nanowires reported so far include the following material combinations: Co/Cu, NiFe/Cu, CoNi/Cu, Ni/Cu, Ni/Au, AgPt, Co/Pt, and Fe/Cu [107–109]. In addition metal–semiconductor–metal junctions, such as Ni–ZnO–Ni and CdTe–Ni, were electrodeposited [110].

#### 2.7 Cap morphology as an indication of wire crystallinity

**Figure 11:**
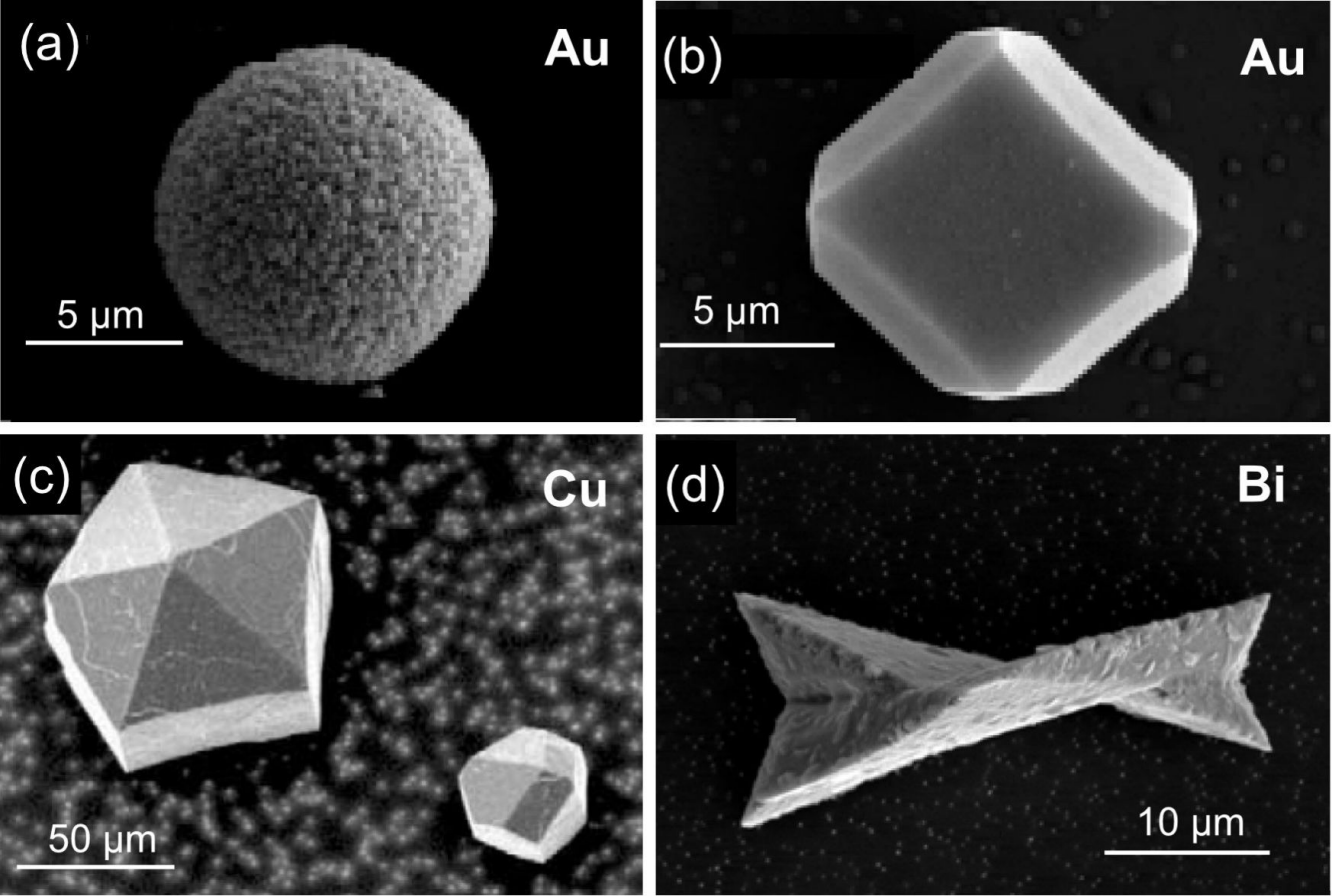
SEM images revealing the characteristic morphology of various metallic caps: (a) polycrystalline Au, (b) single-crystalline Au, (c) twinned Cu and (d) twinned Bi. (a,b) Adapted with permission from [54] – Copyright Springer Verlag 2006; (c) adapted with permission from [71] – Copyright 2005 Elsevier Science Ltd.; (d) adapted with permission from [56] – Copyright 2005 IOP Publishing Ltd.

When the grown nanowires reach the top side of the porous membrane, the deposition continues outside the pores forming so-called caps (Figure 6a, zone 3). The shape and morphology of the caps are a direct indication of the crystalline structure of the wires as shown for various materials (e.g., Cu, Au, Bi, Sb). Round caps are typically formed on top of polycrystalline wires (Figure 11a), while facetted caps grow on top of single-crystalline wires, or on wires consisting of large grains (Figure 11b–d). The facetted Au caps (Figure 11b) exhibit a cubic shape, revealing the cubic structure of the corresponding Au wires. In the case of Cu (Figure 11c), the caps often exhibit a five-fold symmetry. Such morphology is ascribed to multiple twinned crystals consisting of five deformed tetrahedral subunits. The morphology of the facetted Bi caps (Figure 11d) also reveals twinning.

### 3 Nanowire morphology

**Figure 12:**
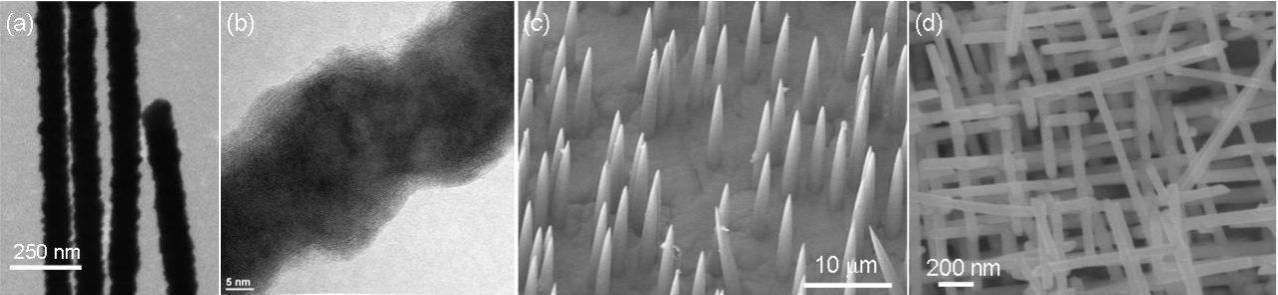
(a) SEM and (b) TEM images displaying the rough contour of Sb nanowires electrodeposited in PET membranes. (c) Array of freestanding conical Cu nanowires. (d) Network of interconnected Sb nanowires. Adapted with permission from [60] – Copyright 2011 American Chemical Society.

The morphology of nanowires, including their geometry, size, and surface contour, is primarily determined by the shape of the hosting channels. The production of templates with swift heavy-ion beams in combination with track etching enables us to control several template parameters such as well-defined channel shape and channel geometry, with the diameter adjustable between a few nanometres up to micrometres, membrane thickness up to 100 μm, and aspect ratios up to 1000. In addition, by varying the fabrication steps in a controlled manner, novel structures can be synthesized, such as pores with conical geometry or channels with smooth or rough inner walls. By exposing the samples to the ion beam under various tilting angles, nanochannel networks with controlled density and interconnection degree are possible. Figure 12 displays a selection of wire morphologies and wire arrangements recently obtained by ion-track technology at GSI: (a,b) rough nanowires; (c) conical nanowires [111]; and (d) nanowire networks.

#### 3.1 Surface roughness

Smooth cylindrical nanowires are suitable for many applications, including sensing of electrical or optical signals. However, in some cases an increased surface roughness is of interest. Hochbaum et al. and Boukai et al. recently reported that rough Si nanowires exhibit a thermal conductivity up to 100 times smaller than their smooth counterparts, becoming promising objects to be implemented in thermoelectric devices [87–88]. In addition, a larger roughness effectively increases the available surface, and thus can be an important advantage, e.g., for catalytic and photovoltaic applications. We have been able to tune the surface roughness of the electrodeposited nanowires by selecting suitable polymer membranes: etched tracks in PC result in smooth channel walls, while channels in PET have a rough surface. Figures 12a and 12b display two exemplary nanowires with diameter 90 and 30 nm, respectively, deposited in PET channels. The variations in diameter along the wire axis amount to 30% in the case of 30 nm diameter wires [60].

#### 3.2 Conical shape

Arrays of nanostructures of cylindrical and conical geometry are promising electrodes for field emission, photovoltaic applications, water splitting, or surface-enhanced Raman spectroscopy [112–113]. The individual cones potentially combine the advantages offered by the reduced dimensions of the tip with the enhanced mechanical stability provided by the large base [114]. An array of freestanding conical Cu wires with a large base of 1–3 µm and a small tip of a few tens of nanometres is shown in Figure 12c. As mentioned above, the apex angle of the cones is determined by the geometry of the hosting conical channel (cf. section 2). Different electrodeposition conditions were studied in order to obtain mechanically stable nanocones with good electrical contact to the substrate. Electrodeposition by using a CuSO_4_-based electrolyte in a two electrode configuration, with *U* = −40 mV leads to a slow growth rate, resulting in a large uniform array of mechanically stable Cu cones of about 28 μm length, 1.2 μm base radius, and 190 nm tip radius [115]. The field-emission properties of similar Cu-nanocone cathodes were investigated by using a field emission scanning microscope (FESM) under ultrahigh vacuum conditions [116]. The improved mechanical stability and solid contact interface of the copper nanocones resulted in much higher emission current values as compared to all previously tested metallic cylindrical nanowires [115,117].

#### 3.3 Nanowire networks

Implementation of nanowire cathodes in fields such as energy harvesting, sensing, or catalysis requires a successful assembly of the nanostructures into 2-D and 3-D architectures [118–119]. Fabrication of 3-D nanowire superstructures by vapour–liquid–solid processes has been reported; however, revealing a limited tunability of the relevant parameters. Recently, Rauber et al. demonstrated the fabrication of highly ordered Pt nanowire networks, consisting of well-defined interconnected nanowires with controlled morphology [120]. Ion irradiation of polymer foils at several incident angles in consecutive steps, followed by chemical etching results in novel etched ion-track membranes with nanochannel arrays tilted at various angles. Electrodeposition in the nanochannel network results in highly ordered 3-D nanowire ensembles. An example of a complex Sb nanowire network is presented in Figure 12d.

#### 3.4 Nanogap structures

**Figure 13:**
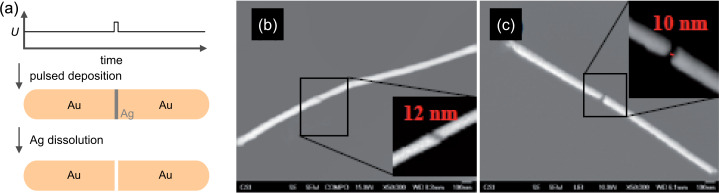
(a) Schema of the electrodeposition and gap-forming process. SEM images of (b) Au-rich/Ag-rich/Au-rich nanowire, and (c) Au nanowire dimer exhibiting a 10 nm gap after selective Ag dissolution. The corresponding insets show details of the Ag segment and the gap, respectively.

Novel nanowire dimer and nanogap structures are interesting for applications in plasmonic sensing as well as nanoelectronics [121–122]. However, the reliable fabrication of such structures remains a challenge. Techniques such as break-junction techniques and gap narrowing by electroplating have been employed, but their precision and reproducibility is limited [123–124]. The template technique offers a promising approach by sequential deposition of multi-material-segmented nanowires grown in porous templates followed by selective etching of one of the deposited elements. This variation of the template method is also known as “on-wire lithography” [125]. Recent experiments yielded segmented Au-rich/Ag-rich/Au-rich nanowires, synthesized by sequential potentiostatic deposition using an electrolyte containing both [Ag(CN)_2_]^−^ and [Au(CN)_2_]^−^ ions [126]. The duration of the sequential pulses controls the length of the silver-rich and gold-rich segments. After dissolution of the polymer membrane, the wires are dispersed in isopropanol. The solution containing the segmented wires is drop-cast onto a substrate. In a subsequent step, the substrate is dipped into concentrated nitric acid to dissolve the silver segments of the wires selectively. The generation of gaps with sizes between 7 and 30 nm is demonstrated [127]. Figure 13 displays schematically the fabrication process (Figure 13a) and shows SEM images of Au nanowire dimers before (Figure 13b) and after (Figure 13c) silver dissolution.

### 4 Size-dependent properties

Nanowires synthesized by electrodeposition in etched ion-track membranes of a large variety of metal, semiconductor and semimetals, whose morphological and crystallographic characteristics can be adjusted in a controlled way as presented above, constitute ideal objects for the investigation of both finite- and quantum-size effects. Due to its technological relevance, size effects on the optical, electrical, and thermal properties are of special interest. The following section presents recent results by the GSI group on electrical, optical, and thermal size effects of electrodeposited nanowires.

#### 4.1 Finite-size effects in electrical properties

Systematic investigations of the electrical transport properties of metal and semiconductor nanowires are necessary in order to better understand classical size effects such as electron scattering at surfaces and grain boundaries. These effects lead to an increase of the specific resistivity of the wire under study compared to its bulk counterpart, which is relevant to nanowire applications such as field-effect transistor sensors, and interconnectors. The influence of grain boundary scattering on the resistivity was predicted decades ago by Mayadas and Shatzkes, and depends on parameters such as electron mean free path, average grain size, and a reflection coefficient at the grain boundaries [128]. The effect of surface scattering was predicted by Dingle et al., and is influenced by nanowire diameter and the specularity of scattering processes at the wire surface [129].

Absolute measurements of the specific resistivity of nanowires require contacting individual nanowires in a reliable manner. The production of low-resistance contacts between nanostructures and macroscopic electronics is a difficult and challenging task. In the case of nanowires, several techniques have been already reported using, for example, the metal-coated tip of a scanning force microscope, optical and electron beam lithography, or manipulators [130–132]. In the case of electrodeposited nanowires, most groups reported the production of large arrays of wires and the subsequent selection of individual wires being contacted with lithographic techniques. Electrical resistivity of individual lithographically contacted Cu nanowires monitored over many hours revealed the critical problem of oxidation. During the measurement, the wire resistance increased from a few hundred ohms to several megaohms. Due to oxidation, the nanowire characteristics change from the metallic to the semiconducting regime [130].

**Figure 14:**
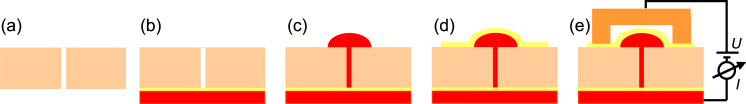
(a–e) Schematic of the fabrication and contacting process for a single nanowire.

Another possibility for contacting single nanowires is based on single-ion irradiation of polymer foils [51]. The single-wire fabrication and contacting process is schematically presented in Figure 14. The steps include (a) the fabrication of a single-pore membrane by ion irradiation and etching; (b) deposition of a suitable substrate; (c) electrochemical growth of a single nanowire (e.g., Cu, Au, Bi) and continuation of the deposition process until a micrometre-sized cap grows on top of the wire; and (d) contacting of the embedded nanostructure by sputtering a conductive layer on the membrane surface. The process avoids the delicate handling of the nanowires and thus minimizes the risk of mechanical damage. Systematic resistivity measurements were performed with single Bi and Au nanowires of various diameters ranging between 40 nm and 1 μm [133–135].

**Figure 15:**
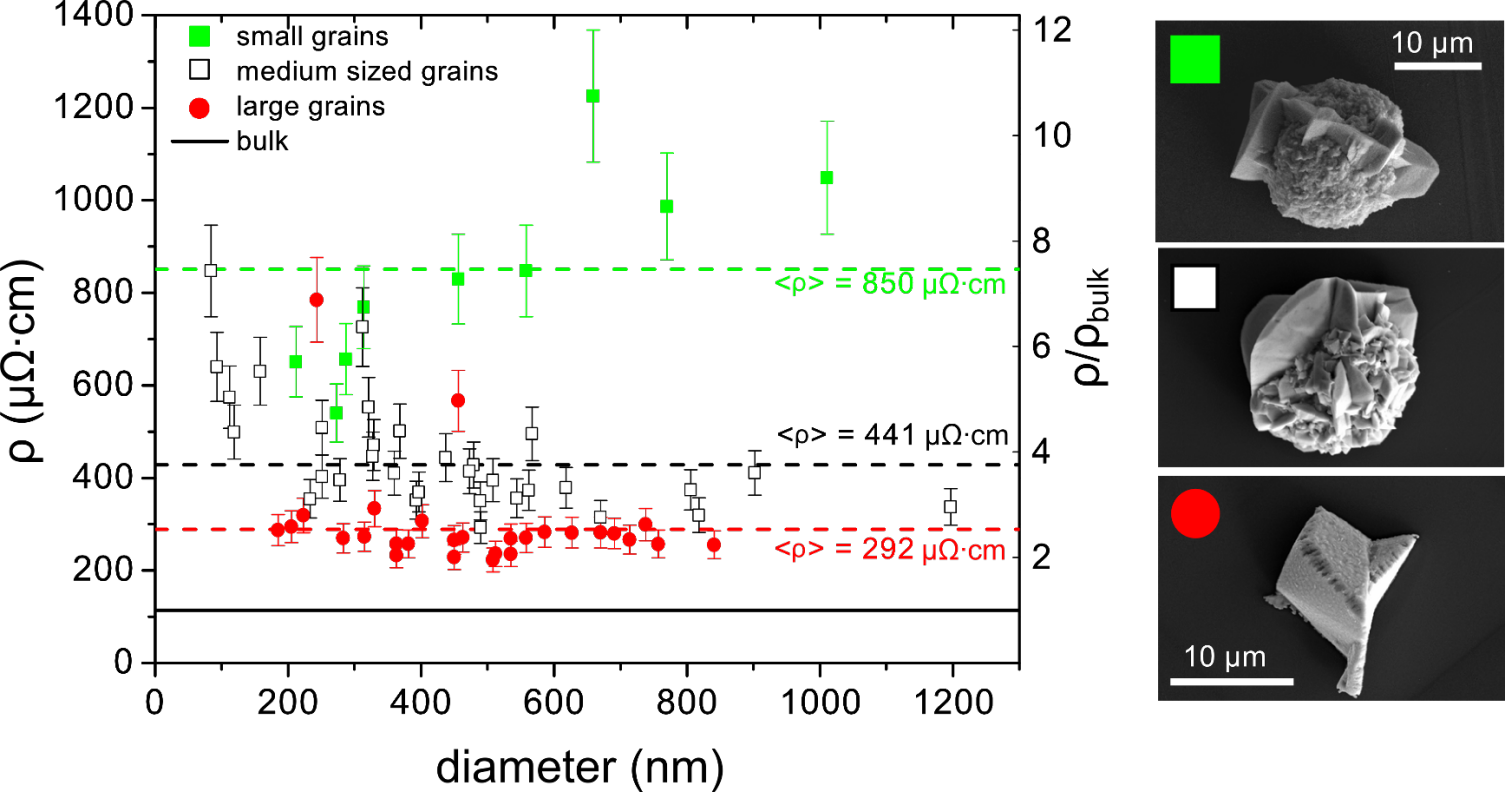
Specific electrical resistivity as a function of diameter of single bismuth nanowires fabricated under three different deposition conditions (*T* and *U*): 30 °C and −50 mV (green squares); 50 °C and −25 mV (open black squares); 60 °C and −17 mV (red circles). The solid line represents the classical behaviour, while the dotted lines are the average resistivity values for the indicated deposition conditions. SEM images illustrate the corresponding cap morphology. Adapted with permission from [133] – Copyright 2006 American Institute of Physics.

**Figure 16:**
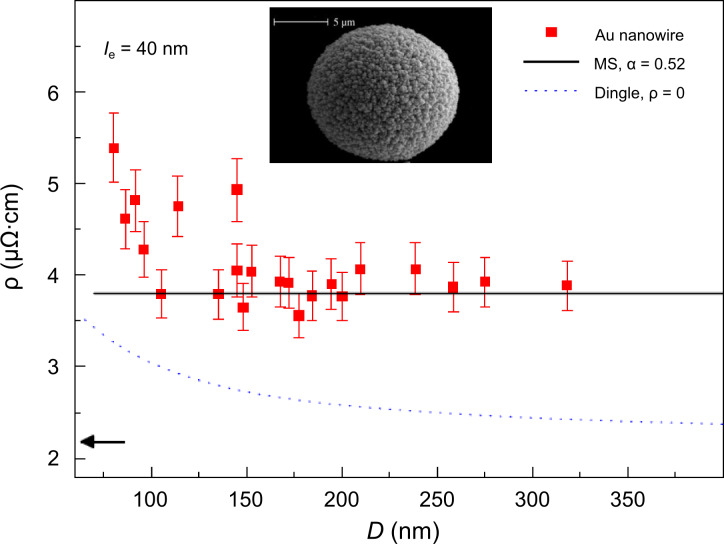
Specific electrical resistivity versus wire diameter for single polycrystalline gold nanowires (ammonium gold(I) sulfite electrolyte, *T* = 50 °C, *U* = −0.8 V) together with the cap morphology. The symbols represent the data. The continuous and dashed lines are a fit of the resistivity predicted by the Mayadas and Shatzkes and the Dingle models, respectively. Adapted with permission from [135] – Copyright 2008 Elsevier B.V.

Figure 15 and Figure 16 display the specific electrical resistivity of individual nanowires as a function of the nanowire diameter for Bi and Au, respectively. In the case of Bi, the electrochemical growth was performed by using three different sets of deposition voltage and temperature. Each of these three wire groups had, thus, a common mean grain size. Figure 15 displays the electrical resistivity of individual Bi nanowires with diameters ranging between 150 nm and 1 µm, which were fabricated by electrochemical deposition in single-pore PC membranes [133].

By using three different sets of deposition voltage and temperature, three groups of single Bi wires could be deposited, each group being characterized by a common mean grain size. The results demonstrate that the resistivity of nanowires with diameters well above 100 nm can be up to eight times higher than for the bulk. For a given diameter, the wire resistivity becomes larger with diminishing grain size due to electron scattering at grain boundaries, providing evidence that the resistivity of nanostructures can be tailored by a suitable choice of the crystalline properties. In the case of Au, all wires show a higher specific resistivity than bulk gold (Figure 16). For diameters larger than 100 nm, the resistivity is nearly constant, with an average value 1.8 times larger than the bulk value, attributed as well to grain-boundary scattering. For *d* < 100 nm the resistivity increases with decreasing diameter, due to additional scattering at the wire surface [135].

Resistance-versus-temperature curves revealed linear characteristics down to 70 K in the case of Au and a nonmonotonic behaviour in the case of Bi [133,135]. The special configuration of a single nanowire embedded and contacted in single nanopore membranes also allows the determination of the maximum current density before failure. Embedded Bi nanowires were found to withstand three to four orders of magnitude higher current densities than bulk Bi. The fact that thinner Bi nanowires can carry higher current densities is attributed to more efficient heat dissipation to the surrounding polymer matrix [136]. For Bi nanowires with a diameter comparable to the Fermi wavelength (i.e., *d* ≈ 100 nm) theoretical calculations predicted that the charge-carrier confinement leads to splitting of the energy bands into sub-bands, and a shift of conduction and valence bands with respect to each other. Such a quantum-size effect was experimentally observed by infrared spectroscopy, revealing a shift of the absorption edge to higher energies with decreasing wire diameter [137].

#### 4.2 Surface plasmon resonances in Au nano-antennas

Collective charge-density oscillations due to surface plasmons exhibited by metal nanoparticles, and in particular by Au nanowires and nanowire dimers (two nanowires separated by a small gap), are being investigated with great interest due to the strongly enhanced electromagnetic fields formed at the nanowire tips and at the gap [123,138]. These surface plasmon resonances (SPRs) are characteristic of each particle and depend on material, dielectric constant of the surrounding medium, and geometry [65–66]. By controlling the length of the nanowire, antenna nanostructures exhibiting SPR at a given frequency of interest can be synthesized. Suitable Au and Cu nanoantennas with specific micrometre length and about 100 nm diameter were fabricated by electrodeposition in PC etched ion-track templates. After dissolution of the polymer membrane, the nanowires were transferred onto infrared (IR)-transparent substrates. Single nanowires were studied at the synchrotron light source ANKA (Forschungszentrum Karlsruhe), with respect to their antenna-like plasmon resonances [138]. The results showed that the resonances depend not only on length and diameter of the wire but also on the substrate and surroundings. Neubrech et al. demonstrated that the IR vibration signals of one attomol of molecules can be detected with enormous sensitivity when the broadband resonance of the nanoantenna matches the IR active vibration dipoles of the molecules [139]. The application of such single nanoantennas and dimers in fields such as surface-enhanced IR absorption or surface-enhanced Raman scattering requires not only an excellent control over the nanostructure synthesis, but also a fundamental understanding of the near-field characteristics of the antennas.

**Figure 17:**
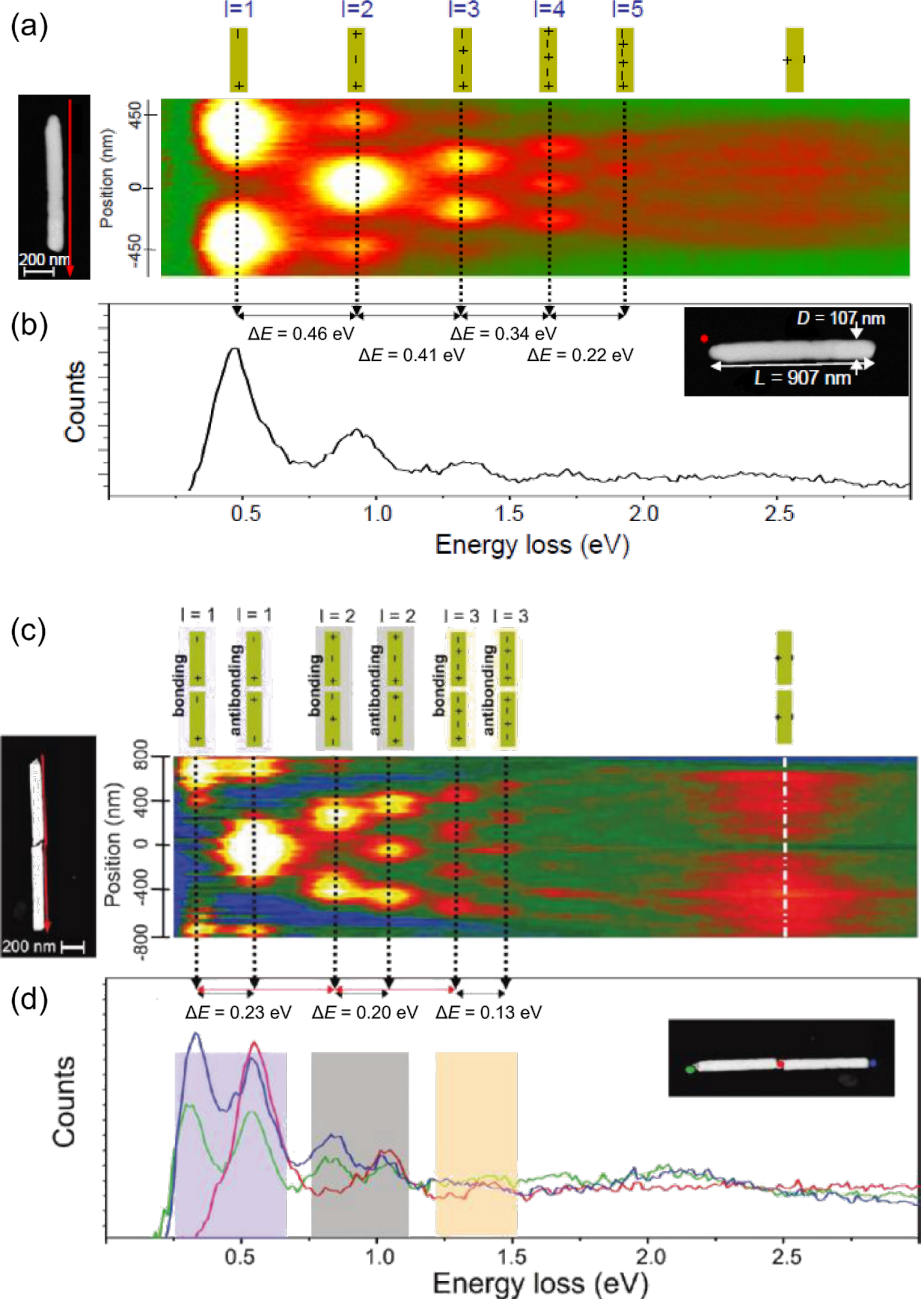
(a) TEM image of the single Au nanowire (length 907 nm, width 107 nm) and the corresponding high-resolution plasmonic field intensity map. The map consists of 50 electron-energy-loss spectra measured along the long axis of the nanowire (cf. direction of arrow next to wire, average distance of scan line to nanowire ≈15 nm). The energy interval, plotted from left to right, ranges from 0.2 to 3.0 eV. The colour indicates the number of counts (white highest). The dotted lines indicate the different multipole modes. (b) Electron energy-loss spectrum measured at one end of the single nanowire (the red dot in the TEM image marks the position of measurement). (c) Plasmonic field intensity map consisting of 60 electron energy-loss spectra measured along a nanowire dimer (red arrow) separated by an ≈8 nm gap. The scan lines have an average distance to the wire of ≈10 nm. The schematics at the top represent the electric-field distribution along the dimer. (d) Electron-energy-loss spectra measured at the two ends of the dimer (blue and green lines) and a spectrum measured in the dimer gap (red line). The coloured dots in the TEM image inset specify the measurement positions for each spectrum. Adapted with permission from [126] – Copyright 2011 American Chemical Society.

Near-field investigations were provided by scanning transmission electron microscopy combined with high-resolution electron-energy-loss spectroscopy (STEM–EELS) using a Zeiss SESAM TEM operated at 200 kV with a field-emission gun and equipped with a MANDOLINE energy filter. This technique allows us to study the transversely and longitudinally localized surface plasmon resonances in single nanowires and nanowire dimers excited by the rapidly travelling electron beam depending on the beam position. Figure 17a shows the high-resolution plasmonic field-intensity map obtained by acquisition from top to bottom (red arrow) of 50 electron-energy-loss spectra at equidistant positions along the long axis of a gold–silver alloy nanowire (*L* = 907 ± 5 nm, *D* = 107 ± 5 nm). The map reveals different plasmon modes, which we assign to the first five longitudinal LSP modes and a transversal mode, and which are schematically presented at the top of Figure 17a. Figure 17b shows a single spectrum of the mapping in Figure 17a, recorded at one side-end of the wire (red dot in TEM picture). Figure 17c displays the plasmonic mapping of a dimer of two nanowires separated by an ≈8 nm gap (*L*_1_ = 784 ± 5 nm, *D*_1_ = 112 ± 5 nm, *L*_2_ = 808 ± 5 nm, *D*_2_ = 112 ± 5 nm). In the case of nanowire dimers, the splitting of the longitudinal multipole modes into bonding and antibonding modes up to the third order (Figure 17c,d) was investigated. Interestingly, the transversal resonance is not excited when positioning the electron beam at the gap and decays rapidly with increasing distance from the wire surface. Figure 17d displays spectra recorded at the dimer ends (blue and green), and at the dimer gap (red). The positions are marked in the TEM image, with dots of the corresponding colours.

#### 4.3 Thermal instability

A successful implementation of nanowire-based devices requires a knowledge of and ability to control the behaviour of the nanostructures at elevated operation temperatures. The thermal stability of nanomaterials is controlled by surface and diffusion processes and influenced by the material characteristics, temperature, and geometrical parameters [140–144]. In particular, it was predicted that nanowires may fragment into a chain of nanospheres above a temperature that is much lower than the corresponding bulk melting temperature *T*_m_. Based on previous theoretical studies by Plateau and Lord Rayleigh on the instability of liquid cylinders and liquid jets [145–146], Mullins and Nichols performed calculations on the thermal instability of solid cylinders considering mass transport by surface and volume diffusion [147]. For a cylinder with initial radius *r* and a sinusoidal perturbation *R* = *r* + Δ*r*_0_ sin(2π*x*/λ), perturbations with wavelength λ > 2π*r* are expected to increase spontaneously in amplitude and become more pronounced with time. The solid cylinder will finally break up into a row of spheres with an average spacing λ_m_ and diameter *d*. Both, λ_m_ and *d* should depend on various factors such as the type of diffusion dominating the transformation, the crystallographic characteristics of the structure, or the surroundings (e.g., the substrate).

**Figure 18:**
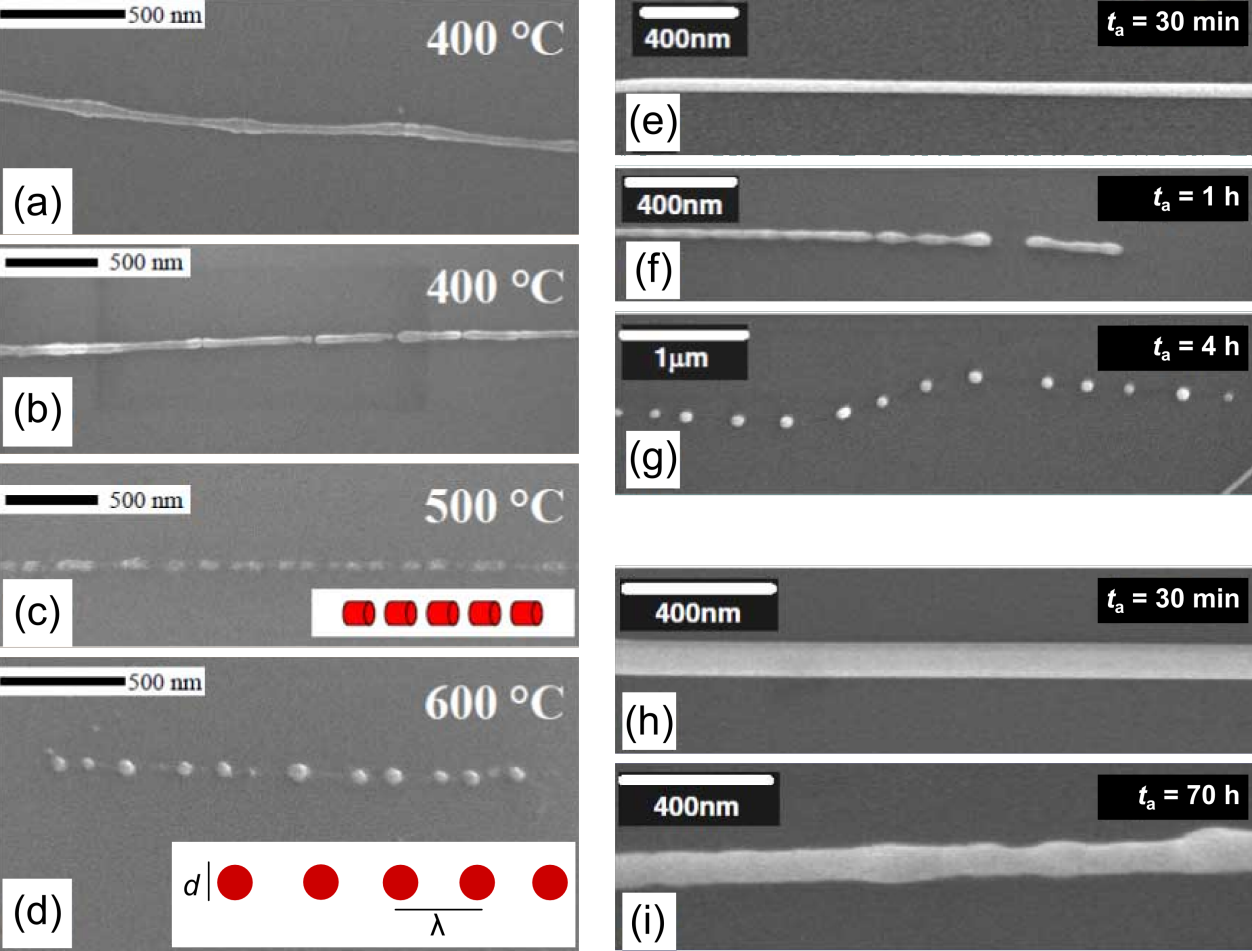
HRSEM micrographs of Cu nanowires of diameter 30 nm after 30 min annealing at different temperatures (a–d), and Au nanowires of two different diameters annealed for various times at 500 °C. The insets represent schematically the fragment geometry, including sphere size and spacing, as modelled by Nichols and Mullins. (e–g) 50 nm diameter Au nanowires after annealing for 30 min (e), 1 h (f), and 4 h (g) at 500 °C. (h–i) 100-nm diameter Au nanowires after 30 min (h) and 70 h (i) annealing. Due to the larger diameter, the wires are more stable and display only soft oscillations after extended annealing. (a–d) Adapted with permission from [148] – Copyright 2004 American Chemical Society; (e–i) adapted with permission from [149] – Copyright 2006 IOP Publishing Ltd.

Systematic thermal annealing experiments studying the morphological transformation of electrodeposited Cu and Au nanowires confirmed that the Rayleigh instability concept is also applicable to metal nanowires [148–149]. In the case of Cu, the fragmentation of nanowires with diameters below 50 nm occurs at temperatures between 400 and 600 °C. HRSEM beautifully visualizes the different stages of the fragmentation process at different temperatures and for different wire diameters (Figure 18a–d).

After annealing at 400 °C, the wire displays diameter fluctuations along the wire axis (Figure 18a) developing into points of fragmentation (Figure 18b). At this temperature the length of the segments is several hundred nanometres. After annealing at 500 °C, the wires decay into shorter sections of length about 100 nm (Figure 18c). Finally at 600 °C, copper nanowires decay into a linear row of spheres (Figure 18d). In the case of Au, nanowires with a diameter of 25 nm develop radial fluctuations already at 300 °C and decay completely into chains of spheres at 500 °C [149]. Figure 18e–g show evidence for the influence of annealing time on the morphological evolution of ≈50 nm diameter nanowires during annealing at 500 °C. For a given temperature, wider nanowires require significantly larger annealing times to induce Rayleigh instability (Figure 18h–i).

The thermal stability is also influenced by the nanowire structure. Single-crystalline Au nanowires oriented along the <110> direction were found to be more stable and required longer annealing times to complete their geometrical transformation into spheres than their polycrystalline counterparts [150]. For both metals, Au and Cu, the final formation of a chain of nanospheres occurs at a temperature much below the melting point (*T*_m_(Cu) = 1083 °C, *T*_m_(Au) = 1064 °C). Recently Zhou et al. reported the fragmentation of Ni nanowires by the Rayleigh criterion at temperatures of about 900 °C (*T*_m_(Ni) = 1453 °C) [151]. This seems to indicate a direct relationship between the bulk melting temperature of the constituent materials and the maximal temperature at which thermal stability is exhibited. These results reveal that prior to nanoscale device applications, technological problems arising from the thermal instability of nanostructures must be seriously taken into account. The positive aspect of the Rayleigh instability is its potential application for converting nanowires into long chains of nanospheres, and the possibility of controlling surface diffusion processes at the nanoscale by applying elevated temperatures.

**Figure 19:**
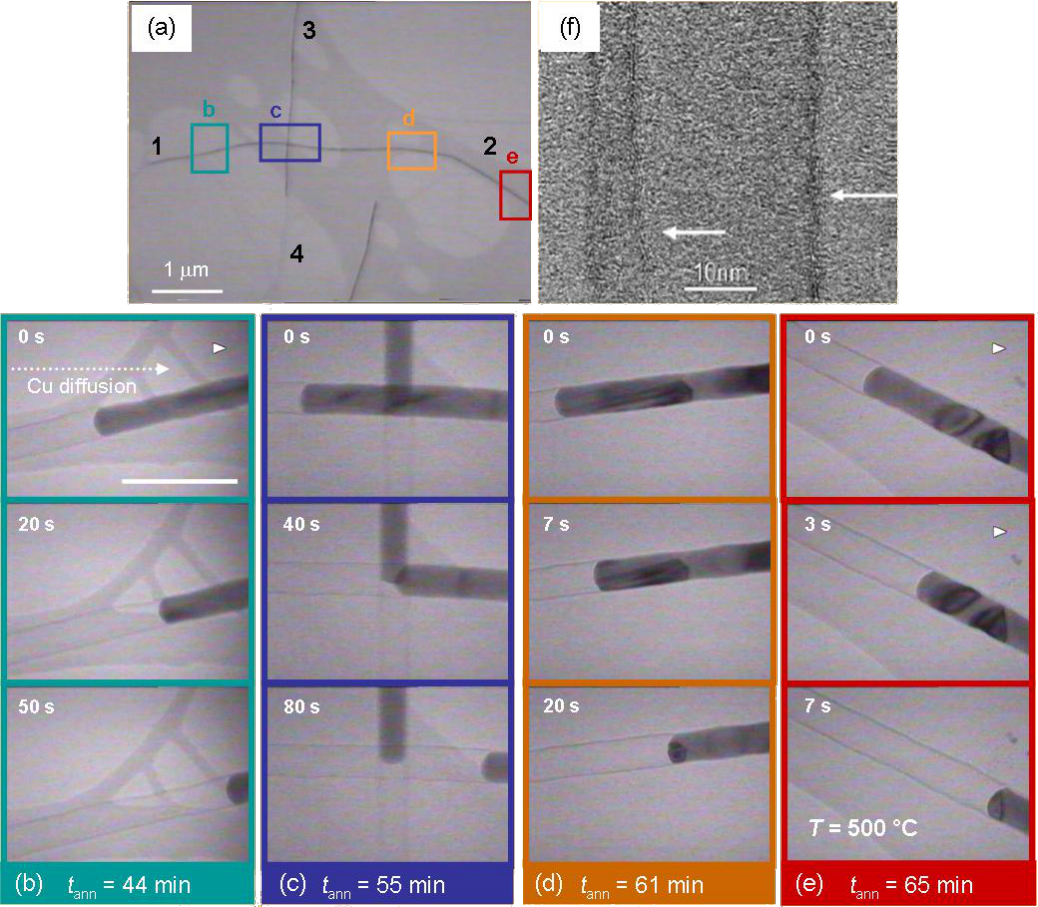
TEM images and video snapshots of Cu nanowire (covered by carbon coating) recorded on four areas marked in (a) at different annealing times: (b) 44, (c) 55, (d) 61, and (e) 65 min. In each case, the time series (from top to bottom) show how the Cu filling moves from left to right, while the carbon shell (f) remains unchanged. The 100 nm scale bar in (a) applies for all snapshots. Adapted with permission from [152] – Copyright 2012 Wiley.

An interesting nanoscale diffusion phenomenon was recently observed for micrometre-long electrodeposited Cu nanowires, confined in a graphitic coating. In situ TEM observation showed that at 500 °C nanowires experience a geometrical transformation into single-crystalline nanoparticles of up to 10-fold increased diameter [152]. Real-time movies recorded in situ visualized the Cu draining out of the carbon coating (Figure 19).

**Figure 20:**
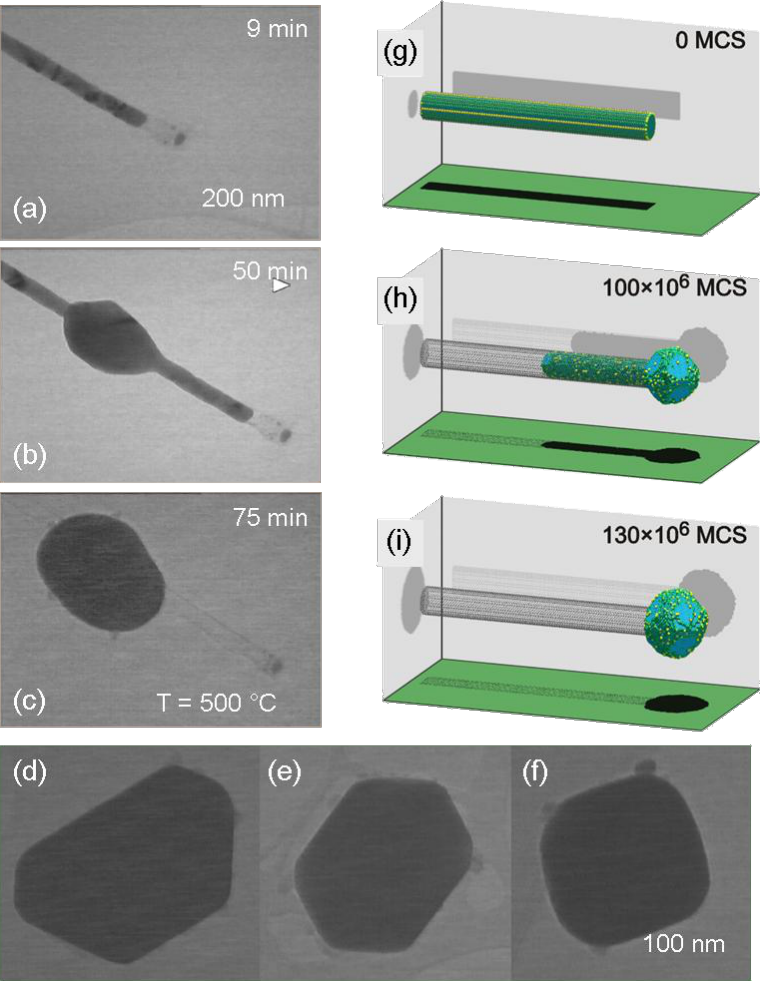
(a–c) TEM images of a Cu nanowire close to the end of a carbon tube (field 2 in Figure 19) visualizing the formation and growth of a nanoparticle during the annealing process. (d–f) Facetted Cu crystals observed at different wire ends after 80 min annealing. (g–h) Snapshots from kinetic Monte Carlo simulations showing the reaction pathway of the draining process. Time is indicated in the number of Monte-Carlo steps (MCS). Adapted with permission from [152] – Copyright 2012 Wiley.

Figure 19a shows a TEM image of two encapsulated Cu nanowires (*d* ≈ 30 nm) intersecting each other on a TEM grid. The wires are covered by a thin carbon layer. The snapshots of the video recorded on the four areas marked in Figure 19a display the effective evacuation of the solid Cu content out of the carbon tube over micrometre distances towards the open end (labelled as 2). The consecutive images of the video (from top to bottom, Figure 19b–e) were recorded at different annealing times (*t*_ann_ = 44, 55, 61, and 65 min). Although the temperature is constant (500 °C), the velocity of the Cu front increases with time and location during the annealing process. Details of this drainage process close to the open end of a carbon tube are shown in Figure 20a–c. Figure 20a–c shows TEM images of the Cu nanowire in Figure 19a, close to the end 2, taken at different times during the annealing process. This series of images visualizes the formation and growth of a nanoparticle during the annealing process. Once the process has been completed, each nanowire is transformed into a single monocrystalline, facetted Cu particle (Figure 20d–f).

Based on kinetic Monte Carlo simulations (Figure 20g–i) it is proposed that this dramatic morphological transformation is driven by surface diffusion of Cu atoms along the wire-tube interface, thus minimizing the total free energy of the system. The high-resolution micrograph displayed in Figure 19f displays the multishell graphitic coating presumably formed from polymer residues on the nanowire surface. It is noteworthy that this carbon layer is resistant throughout the whole process. The formation and characteristics of such fascinating carbon-coated core–shell structures should be investigated in more detail. The results also show that nanowires coated by electron-beam-induced carbon tubes can serve as well-defined nanopipettes. The extraction process is initiated and controlled by temperature. The template-based electrochemical wire synthesis allows control over the wire diameter as well as length and thus provides material for nanocrystals whose size is predefined by the pipette volume.

## Conclusion

We have described the synthesis of micro- and nanowires using ion-track technology in combination with electrodeposition. We illustrate how this technique enables the independent and simultaneous control of size (diameter and length), morphology, crystalline structure, and composition of the nanowires. A combination of irradiation of polymer foils with high-energy heavy ions and chemical etching results in templates with micro- and nanochannels (length of several tens of micrometres and diameter from ca. 10 nm to several micrometres). By electrodeposition in the channels, nanowires of different materials, such as Au, Cu, Pt, Bi, Bi_2_Te_3_, ZnO, and CdTe, are synthesized. The crystallographic characteristics (surface roughness, grain size, and texture) are also well controlled by various electrodeposition parameters, namely voltage, temperature, and electrolyte. Finally, several examples of recent results by the GSI group on electrical, optical and thermal size effects of electrodeposited nanowires have been presented, demonstrating that these electrodeposited nanowires constitute ideal objects for the investigation of both finite- and quantum-size effects.
